# Novel pyripyropenes produced by gene cluster design and heterologous expression

**DOI:** 10.1080/21501203.2025.2565221

**Published:** 2025-09-30

**Authors:** Shunjin Jia, Yuchen Wu, Yihui Chen, Yuangui Tang, Yunlu Cui, Qunjian Yin, Daxiong Ji, Pinmei Wang, Jinzhong Xu

**Affiliations:** a Ocean College, Zhejiang University, Zhoushan, China; b Shenyang Institute of Automation, Chinese Academy of Sciences, Shenyang, China; c College of Atmospheric Sciences, Sun Yat-sen University, Zhuhai, China; d Key Laboratory of Tropical Marine Ecosystem and Bioresource, Fourth Institute of Oceanography, Ministry of Natural Resources, Beihai, China

**Keywords:** Novel pyripyropenes, structural diversity, synthetic biology, heterologous expression, *Aspergillus nidulans*

## Abstract

Post-modification of the isoprenoid ring in pyripyropenes critically modulates their structural diversity and bioactivity. Here, we report the engineered biosynthesis of novel pyripyropene derivatives via reconstruction of the pyripyropene A gene cluster and its heterologous expression in *Aspergillus nidulans*. Five previously unreported compounds—1-keto-pyripyropene E (**1**), 13-hydroxy-1-keto-pyripyropene E (**2**), deacetyl-pyripyropene G (**3**), 11-deoxo-deacetyl-pyripyropene A (**4**), and 1-keto-11-deoxo-deacetyl-pyripyropene A (**5**)—were isolated from the engineered strain. Their structures were unequivocally characterised by MS and NMR spectroscopy, with compounds **1**, **2**, and **5** representing the first examples of 1-ketone pyripyropenes. Significantly, these 1-ketone derivatives exhibited significantly enhanced insecticidal activity compared to their 1-hydroxy analogues, demonstrating their potential as next-generation insecticide leads.

## GRAPHICAL ABSTRACT

**Figure:**
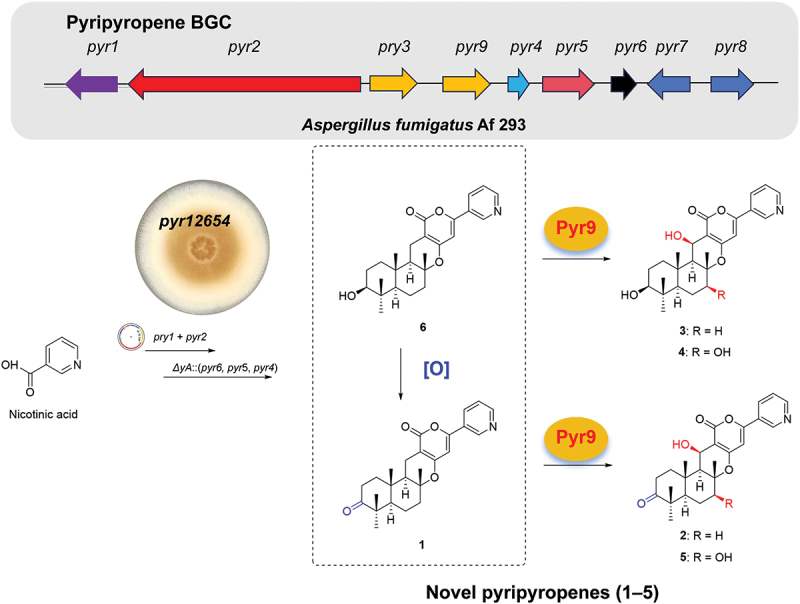

## 1. Introduction

**Figure 1.**
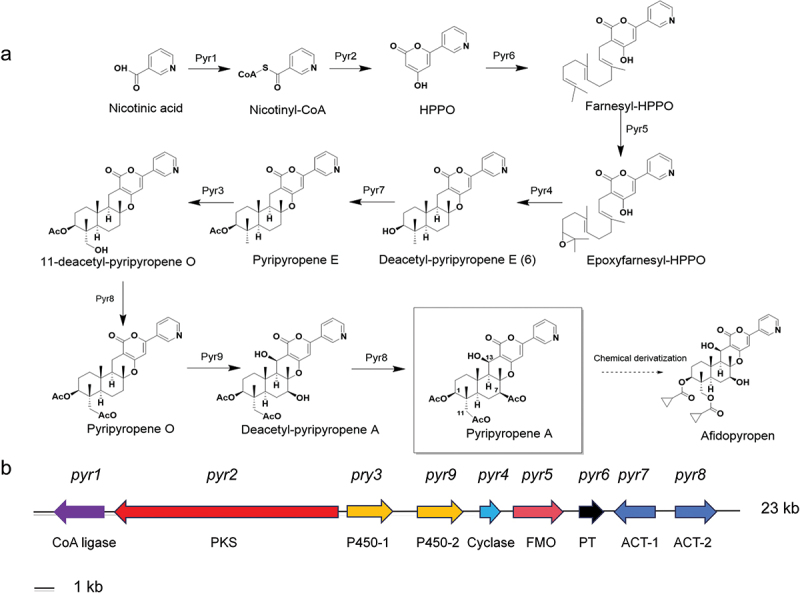
The biosynthetic pathway and gene cluster of pyripyropene A. (a) Proposed biosynthetic pathway of pyripyropene A and chemical derivatization of pyripyropene A to afidopyropen. (b) Biosynthetic gene cluster of pyripyropene A in *Aspergillus fumigatus* Af 293. CoA ligase, coenzyme A ligase; PKS, polyketide synthase; P450, cytochrome P450 monooxygenase; FMO, FAD-dependent monooxygenase; PT, prenyltransferase; ACT, acetyltransferase.

The fungal meroterpenoid pyripyropene A (PP-A) exhibits significant biological activities, including potent inhibition of acyl-coenzyme A: cholesterol acyltransferase (ACAT) (Omura et al. 1993) and high insecticidal activity against aphids (Horikoshi et al. 2017). Building upon this foundation, a semi-synthetic derivative of PP-A named afidopyropen was developed. Afidopyropen features cyclopropanecarbonyloxy groups at the C-1 and C-11 positions and is deacetylated at C-7 (Figure 1a). This optimised compound was launched globally (e.g., in the USA, India, China, and Australia) by BASF as the commercial insecticide Inscalis® (Horikoshi et al. 2022).

Structurally, pyripyropenes (PPs) possess a characteristic meroterpenoid backbone (Figure 1a). This consists of a tricyclic C15-terpenoid moiety fused to a polyketide-derived portion, specifically a pyridine-substituted pyrone ring. Naturally occurring PPs typically contain hydroxyl groups at C-1, C-7, C-11, and C-13, with the hydroxyls at C-1, C-7, and C-11 often being acetylated (Kim et al. 1994; Tomoda et al. 1995, 1996; Itoh et al. 2010; Shan et al. 2015; Chiang et al. 2016; Cao et al. 2017; Ying et al. 2018; Lin et al. 2020; Zou et al. 2022; Chen et al. 2025). The biosynthetic pathways for PPs have been elucidated by identifying their gene clusters in fungi such as *Aspergillus fumigatus* (Itoh et al. 2010) and *Penicillium coprobium* (Hu et al. 2011, 2014) (Figure 1b). Key enzymatic steps involve hydroxylation and acetylation: two cytochrome P450 monooxygenases, Pyr3 and Pyr9, catalyse hydroxylation at specific positions, with Pyr3 hydroxylating C-11 and Pyr9 hydroxylating both C-7 and C-13 (Hu et al. 2011); subsequently, two acetyltransferases, Pyr7 and Pyr8, modify the resulting hydroxyl groups, with Pyr7 acetylating the 1-OH group and Pyr8 acetylating the 7-OH and 11-OH groups (Hu et al. 2014) (Figure 1a).

Leveraging this detailed understanding of the biosynthetic machinery, we employed a synthetic biology strategy to generate structural diversity within the pyripyropene class. By manipulating the identified genes in the heterologous fungal host *Aspergillus nidulans*, we successfully produced five novel pyripyropenes (**1**–**5**). This approach, based on rational design of the pyripyropene biosynthetic gene cluster (BGC), enabled the creation of derivatives with altered hydroxylation and acetylation patterns. Initial characterisation of these compounds provides valuable insights into structure-activity relationships (SAR) and underscores the potential of biosynthetic engineering for discovering novel insecticides.

## 2. Materials and methods

### 2.1. Strains and culture conditions

The strains used in this study are listed in Table S1. *Aspergillus fumigatus* Af 293 containing the pyripyropene biosynthetic gene cluster (Osherov et al. 2001) and the triple auxotrophic chassis strain *Aspergillus nidulans* LO8030 (*pyrG*- *riboB*- *pyroA*-), a powerful heterologous expressing chassis which possesses a minimised endogenous metabolic interference owing to the deletion of eight highly expressed native biosynthetic gene clusters (BGCs) (Chiang et al. 2013, 2016) were maintained as glycerol stocks at −80 °C and activated at 37 °C on solid glucose minimum medium (GMM) (Shimizu and Keller 2001) supplemented as needed for auxotrophs. GMM was supplemented with 0.5 g/L uracil and 0.5 g/L uridine for *pyrG* auxotroph, 1 mg/L pyridoxine HCl for *pyroA* auxotroph, and 2.5 mg/L riboflavin for *riboB* auxotroph. *Saccharomyces cerevisiae* BJ54642 (Yin et al. 2013) was used for plasmid constructions via *in vivo* yeast recombination, and *Escherichia coli* DH5α was used for plasmid amplification.

### 2.2. Genetic manipulations

*A*. *fumigatus* Af 293 and *A. nidulans* were cultured at 37 °C for 24 h in steady liquid GMM medium, supplemented according to auxotrophic requirements. Genomic DNA (gDNA) was then extracted using the standard protocol (Aamir et al. 2015). The biosynthetic gene cluster (BGC) genes for pyripyropene A were PCR-amplified from *A. fumigatus* Af 293 gDNA using the high-fidelity DNA polymerase Primestar HS/MAX (TaKaRa, Japan). All primers used in this study are listed in Table S2. Plasmids (Table S3) were constructed via *in vivo* yeast recombination in *Saccharomyces cerevisiae* BJ5464, as described previously (Wang et al. 2016).

First, the “*E. coli-yeast-Aspergillus*” shuttle plasmid pSJ1 was constructed for the heterologous expression of the coenzyme A (CoA)-ligase gene *pyr1* and the iterative type I polyketide synthase (PKS) gene *pyr2* in *A. nidulans* LO8030. The backbone of pSJ1 contains *ori*, *2µ*, and *AMA1* elements, enabling autonomous replication in *E. coli*, yeast, and *Aspergillus*, respectively. In pSJ1, *pyr1*, and *pyr2* are controlled by the constitutive promoter *gpdAp* (Wang et al. 2016) (from *A. nidulans*) and the starch-inducible *Aspergillus* promoter *amyBp* (Awakawa et al. 2012), respectively. The plasmid also carries the uracil/uridine auxotrophic marker gene *AppyrG* (the *pyrG* gene from *Aspergillus parasiticus*) for selection of correct transformants. *A. nidulans* protoplast preparation and transformation were carried out as described in references (Bok and Keller 2004; Ma et al. 2018). *A. nidulans* transformant harbouring pSJ1 was named SSJ1.

Subsequently, the plasmid pSJ9—containing the cyclase gene *pyr4*, flavin-dependent monooxygenase (FMO) gene *pyr5*, and transmembrane prenyltransferase (PTase) gene *pyr6*- was constructed via the same yeast-based *in vivo* assembly method (Wang et al. 2016). A control transformation cassette was generated by PCR amplification, comprising: the promoters *gpdAp* and *amyBp*, genes *pyr4*, *pyr5*, and *pyr6*, the *Aspergillus fumigatus riboB* auxotrophic marker (*AfriboB*), as well as 1 kb flanking sequences homologous to the upstream and downstream regions of the *yA* locus. This cassette was transformed into SSJ1 protoplasts, while the fragment “*amyBp-pyr4-gpdAp-pyr5-gpdAp-pyr6-AfriboB*” was integrated into the chromosomal *yA* locus through homologous recombination, yielding transformant SSJ9.

Plasmids pSJ10 (containing cytochrome P450 monooxygenase genes *pyr3* and *pyr9*), pSJ12 (containing a double copy of *pyr3*), and pSJ13 (containing a double copy of *pyr9*) were constructed. Using these plasmids as templates, we generated three linear expression cassettes by PCR: 1) *amyBp-pyr3-gpdAp-pyr9-pyroA*, 2) *amyBp-pyr3-gpdAp-pyr3-pyroA*, and 3) *amyBp-pyr9-gpdAp-pyr9-pyroA*. These PCR products were individually transformed into strain SSJ9, targeting integration at the *wA* locus. This yielded three distinct pyripyropene heterologous expression transformants: SSJ10, SSJ12, and SSJ13.

### 2.3. Fermentation and HPLC/MS analysis

To compare secondary metabolite profiles across *A. nidulans* transformants, strains SSJ9, SSJ10, SSJ12, SSJ13, and the control LO8030 were fermented in liquid GMM medium. The medium was supplemented with 20 g/L starch (to induce biosynthetic gene expression) and 1 g/L nicotinic acid (a pyripyropene precursor). Due to auxotrophy, SSJ9 cultures additionally received 1 mg/L pyridoxine HCl. Fermentations were performed in triplicate using 500 mL shake flasks containing 200 mL medium, incubated at 30 °C and 180 r/min for 7 d. Secondary metabolites were subsequently extracted from all cultures.

After fermentation, mycelia were harvested by filtration. The harvested mycelia underwent ultrasonic-assisted extraction (Xiao et al. 2008): The mycelia were suspended in 100 mL methanol within a 500 mL conical flask and subjected to three 30-minute extraction cycles in a 25 ℃ ultrasonic water bath. Concurrently, the fermentation broth was extracted three times with an equal volume of ethyl acetate. The combined organic phases from both extractions were concentrated under reduced pressure. The resulting residues were dissolved in 1 mL methanol, filtered through a 0.22 μm membrane filter, and analysed by HPLC and LC-MS.

HPLC analysis was performed on the Agilent 1260 HPLC system equipped with an Eclipse Plus C18 column (4.6 × 250 mm, 5 μm). Separation used a linear gradient of 10% to 100% acetonitrile in water acetonitrile containing 0.05% trifluoroacetic acid (TFA) over 60 min, at a flow rate of 1 mL/min, with detection at 322 nm.

LC-MS analysis was conducted using an Agilent 6230 TOF system with electrospray ionisation (positive ion mode) and the same C18 column. The mobile phase consisted of a linear gradient from 10% to 100% acetonitrile in water at a flow rate of 0.4 mL/min.

### 2.4. Purification and structural characterisation of novel compounds

For purification of the novel compounds, transformant SSJ13 was fermented in 10 L medium at 30 °C, 180 r/min for 7 d. After fermentation, the broth was extracted three times with an equal volume of ethyl acetate, while the mycelia underwent triple ultrasonic extraction with 2 L methanol. Combined organic phases were concentrated under reduced pressure to yield 16.20 g crude extract.

The crude extract was fractionated via silica gel column chromatography (CH₂Cl₂/MeOH gradient: 50:1 → 25:1 → 10:1, v/v). Fractions containing pyripyropenes, identified by analytical HPLC, were further purified by preparative HPLC (Ultimate® XB-C18, 21.2 × 250 mm, 5 µm). Compound-specific mobile phases were employed: **1** and **6** used acetonitrile-water (37:63, v/v), **2** used acetonitrile-water (43:57, v/v), **3** used acetonitrile-water (40:60, v/v), **4** used methanol-water (50:50, v/v), and **5** used methanol-water (55:45, v/v), all at 10 mL/min flow rate.

Purified compounds were dissolved in CDCl₃ or DMSO-*d*₆ according to solubility requirements. Structural characterisation was performed on a JEOL 600 MHz NMR spectrometer, acquiring ^1^H NMR, ^13^C NMR, and 2D spectra (HSQC, HMBC, ^1^H-^1^H COSY, NOESY).

### 2.5. *Insecticidal activity assay against* Aphis craccivora

Compounds **1**–**5** (purified from microbial extracts) and afidopyropen standard were tested for insecticidal efficacy against cowpea aphid (*Aphis craccivora*) using a live plant spray bioassay (Horikoshi et al. 2017). Broad bean (*Vicia faba*) seedlings were cultivated individually in 7-cm pots until the first true leaves expanded. Apical buds were excised prior to infestation with 30–50 synchronised 2-day-old aphid nymphs per plant. After 24 h stabilisation, initial nymphal counts were recorded. Test compounds were dissolved at serial concentrations and sprayed uniformly onto leaf surfaces (2.5 mL/pot) using a laryngeal atomiser. Treated plants were air-dried at room temperature, with three biological replicates per concentration. Aphid mortality was assessed 96 h post-treatment. Corrected mortality (%) was calculated as:

$$\mathrm{Mortality}=\;\frac{\mathrm{Initial}\;\mathrm{nymphs}-\mathrm{Surviving}\;\mathrm{nymphs}}{\mathrm{Initia}\;\mathrm{lnymphs}}\times 100$$

## 3. Results and discussion

**Figure 2.**
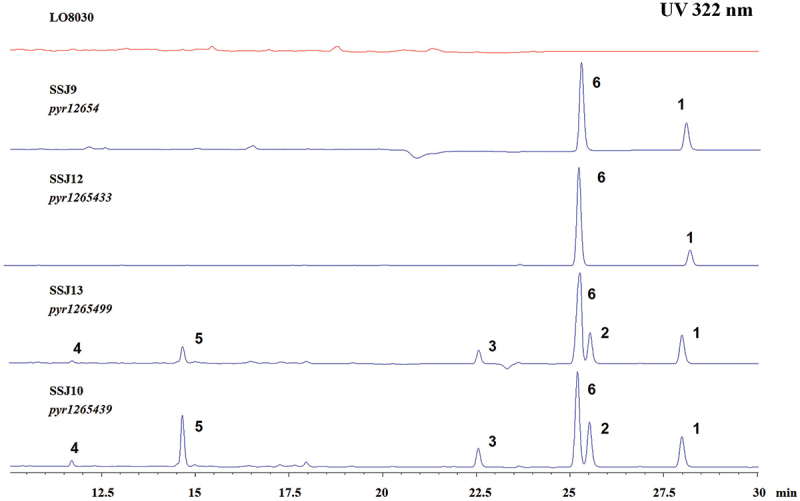
HPLC analysis for metabolites from LO8030, SSJ9, SSJ12, SSJ13, and SSJ10.

According to the predicted functions of genes within the biosynthetic gene cluster (BGC) and the elucidated pyripyropene A (PP-A) pathway (Figure 1a), we co-expressed five genes in *Aspergillus nidulans* LO8030 (Itoh et al. 2010), including *pyr1* encoding the coenzyme A (CoA)-ligase, *pyr2* encoding the iterative type I polyketide synthase (PKS), *pyr6* encoding the transmembrane prenyltransferase (PTase), *pyr5* encoding the flavin-dependent monooxygenase (FMO), and *pyr4* encoding the cyclase, to try to produce deacetylated PPs. The resulting strain SSJ9 (expressing *pyr12654*) produced deacetyl-pyripyropene E (**6**), the simplest PP derivative, detected by HPLC (λ = 322 nm; characteristic of the pyrone-pyridine ring) (Figure 2). Unexpectedly, an additional compound (**1**) co-occurred with **6** in SSJ9 fermentations. Subsequently, the cytochrome P450 monooxygenase genes *pyr3* and *pyr9* were individually introduced into SSJ9 to generate hydroxylated derivatives. While transformant SSJ12 (expressing *pyr3*) yielded only **1** and **6** (Figure 2), both SSJ10 (expressing *pyr3* and *pyr9*) and SSJ13 (expressing *pyr9*) produced six PP metabolites (**1**–**6**) with identical HPLC profiles (Figure 2).

**Figure 3.**
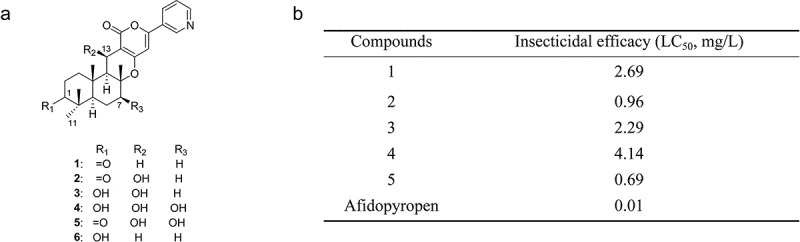
The structures of PPs in this study and their insecticidal efficacy. (a) The structures of compounds **1**–**6**. (b) Insecticidal efficacy of **1**–**5** against *Aphis craccivora*.

The transformant SSJ13 was subjected to large-scale fermentation (10 L) for the isolation of pyripyropene (PP) derivatives. Combined silica gel column chromatography and preparative ODS-HPLC yielded pure compounds: **1** (20 mg, ACN/H_2_O 37:63), **2** (12.8 mg, ACN/H_2_O 43:57), **3** (21.4 mg, ACN/H_2_O 40:60), **4** (17.7 mg, MeOH/H_2_O 50:50), **5** (20.5 mg, MeOH/H_2_O 55:45), **6** (120 mg, ACN/H_2_O 37:63) (Figure 3a).

**Figure 4.**
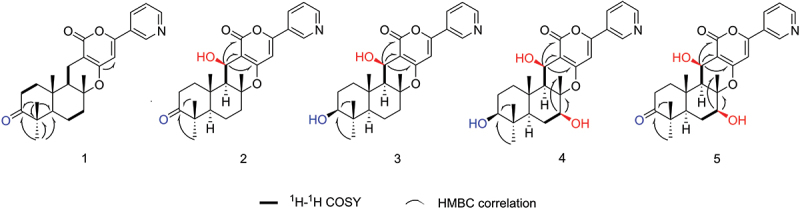
The key ^1^H-^1^H COSY and HMBC correlations of compounds **1**–**5**.

Compound **1**: white powder. The UV (MeOH) λ_max_ of 230 and 322 nm showed the presence of pyridyl-pyrone, a typical chromophore of pyripyropenes. TOF-ESI-MS (Figure S2) gave out the ions at *m/z* 408.2168 for [M+H]^+^, *m/z* 430.1985 for [M+Na]^+^, and *m/z* 837.4087 for [2 M+Na]^+^, respectively, and indicated the molecular formula of C_25_H_29_NO_4_ (calcd. 408.2169 for C_25_H_30_NO_4_), two hydrogen atoms less than deacetyl-pyripyropene E (**6**) (Itoh et al. 2010). Comparison of the ^13^C NMR between compounds **1** and **6** (Table S4) showed that an additional ketone carbonyl carbon signal at *δ*_C_ 216.0 in data of **1** replaced the oxygenated carbon signal at *δ*_C_ 78.4 for C-1 in **6**, which indicated compound **1** was the ketone derivative at C-1 of deacetyl-pyripyropene E (**6**). The key HMBC correlations (Figure 4) from CH_3_-11 (*δ*_H_1.14, s) and CH_3_-15 (*δ*_H_1.08, s) to C-1 (*δ*_C_ 216.0) confirmed this deduction. **1** was the first 1-ketone derivative of pyripyropenes and named as 1-keto-pyripyropene E. 1D-NMR data of **1** were assigned (Table S4) through the detailed analysis of 2D-NMR spectra (Figure S2).

Compound **2**: white powder. TOF-ESI-MS (Figure S3) gave a series of adduct ions at *m/z* 424.2112 for [M+H]^+^, *m/z* 446.1944 for [M+Na]^+^, and *m/z* 869.3982 for [2 M+Na]^+^, respectively, indicating the molecular formula of C_25_H_29_NO_5_. Its molecular weight is 16 Da greater than **1** which determined **2** as the oxidative product of **1**. The noticeable proton signal (*δ*_H_ 5.00, d, *J* = 4.2 Hz) and carbon signal (*δ*_C_ 60.4) were observed in the 1D-NMR data of **2**, and then these signals were assigned to CH(OH)-13 by the ^1^H-^1^H COSY correlation between H-13 (*δ*_H_ 5.00) and H-5 (*δ*_H_ 1.56), together with HMBC correlations from H-13 (*δ*_H_ 5.00) to C-2’ (*δ*_C_ 164.2), C-3’ (*δ*_C_ 103.5), C-4’ (*δ*_C_ 162.7), C-5 (*δ*_C_ 55.6), C-6 (*δ*_C_ 82.0) (Figure 4). The other NMR signals were similar to those of **1** (Table S4 and S5). Then the structure of **2** was elucidated as 13-hydroxyl derivative of **1** and named as 13-hydroxyl-1-keto-pyripyropene E, which was confirmed by the 2D-NMR analysis. Structural comparison with the reported pyripyropenes showed that **2** was identical to pyripyropenes G-H obtaining 13-OH and no 7/11-OH (Tomoda et al. 1995). The orientation of 13-OH was determined as *β* according to the coupling constant of 4.2 Hz (Table S5).

Compound **3**: white powder. The molecular formula was determined as C_25_H_31_NO_5_ by the mass spectral data (Figure S4) such as *m/z* 426.2268 for [M+H]^+^, *m/z* 448.2089 for [M+Na]^+^, and *m/z* 873.4308 for [2 M+Na]^+^. The molecular weight is 2 Da greater than **2** and 16 Da greater than **6**. The carbon NMR of **2** showed noticeable disappearance of ketone signal. Comparing the ^13^C NMR spectra of compounds **2**, **3**, and **6** (Figure S1) elucidated **3** as oxidative product of **6** with an additional hydroxyl positioned at C-13, similar to compound **2** (Figure 3a). The structure of **3** was finally identified as deacetyl-pyripyropene G by key HMBC correlations from H-13 (*δ*_H_ 4.77) to C-2’ (*δ*_C_ 162.3), C-3’ (*δ*_C_ 103.4), and C-4’ (*δ*_C_ 162.1) (Figure 4).

Compounds **4** and **5** were also obtained as white powder. The TOF-MS data (Figure S5 and S6) of **4** and **5** showed 16 Da greater than **3** and **2**, respectively, indicating that the formers were the oxidative products of the latters. The characteristic signals (*δ*_H_ 3.58, *δ*_C_ 76.5 in **4**; *δ*_H_ 3.63, *δ*_C_ 75.6 in **5**) (Table S6) for CH-OH groups were observed in their NMR spectra and the CH-OH were positioned at C-7 by the HMBC correlations from CH-7 to C-6 and C-14 (Figure 4). Compared with reported pyripyropenes, CH_3_-11 in **4** and **5** lacked hydroxylation. Then compounds **4** and **5** were named as 11-deoxo-deacetyl pyripyropene A and 1-keto-11-deoxo-deacetyl pyripyropene A, respectively.

The 1-keto-PPs have never been reported previously while three compounds **1**, **2**, and **5** were firstly discovered in this work. Short-chain dehydrogenase/reductases (SDRs), such as OlcF have been reported to transform 27-hydroxyl of decaturins E and F to a ketone group (Yaegashi et al. 2015). Therefore, we proposed that the substrates **6**, **3**, and **4** were catalysed by some SDR in the chassis strain *A. nidulans* to be **1**, **2**, and **5**, respectively (Table S7).

The comparison of the metabolite profiling of strains SSJ9, SSJ10, SSJ12, and SSJ13 revealed that P450 Pyr3 exhibits strict substrate selectivity, specifically catalysing the modification of 1-O-acetoxyl PPs. This result further corroborates the findings from heterologous expression and substrate co-incubation assays (Hu et al. 2011). In contrast, P450 Pyr9 demonstrated catalytic promiscuity, capable of modifying diverse functional groups, including 1-hydroxyl (**6**), 1-keto (**1**), and 1-acetoxy (PP-O) (Hu et al. 2011), an activity not previously characterised.

The insecticidal efficacy of new compounds **1**–**5** was tested against *Aphis craccivora* (Figure 3b). The anti-aphid activities of compounds **2** and **5** were comparable to that of pyripyropene A, which has an LC_90_ value of 0.56 ppm (Goto et al. 2019a, 2019b). Studies on the relationship between the insecticidal activity of PPs and their modifying groups have indicated (Goto et al. 2018, 2019a) that hydroxylation at C-7 and C-13 plays a crucial role in their insecticidal efficacy. The experimental results revealed that the insecticidal activity of compound **5** was higher than that of compound **2**, which in turn was higher than that of compound **1**. This observation confirms that hydroxylation at C-7 and C-13 is indeed critical for the activity of PPs. The insecticidal activities of compounds **2** and **5** were significantly higher than those of PPs hydroxylated at the C-1 position (compounds **3** and **4**). Although compound **1** only features ketonisation at the C-1 position without 7-OH or 13-OH modifications, its insecticidal activity was still comparable to that of compounds **3** and **4**. These findings collectively demonstrate that the introduction of a 1-ketone group significantly enhances the insecticidal activity of PPs.

## 4. Conclusions

In conclusion, heterologous expression of a designed pyripyropene biosynthesis gene cluster in the fungal chassis host *A. nidulans* LO8030 yielded five novel pyripyropene derivatives. This study reports the first isolation of 1-ketone pyripyropenes, which demonstrated significantly stronger insecticidal activity than their 1-hydroxyl analogues. These findings underscore the potential of 1-ketone functionalization in developing next-generation pyripyropene-based insecticides.

## Supplementary Material

Supplemental Material
