# Supplementary material for: Novel pyripyropenes produced by gene cluster design and heterologous expression
Source: Mycology. 2025 Sep 30;17(2):543–51. doi: 10.1080/21501203.2025.2565221 (PMC13267016; doi:10.1080/21501203.2025.2565221)
Supplement: Supplemental Material [file TMYC_A_2565221_SM6721.docx]

**Supplemental material**

**Novel pyripyropenes produced by gene cluster design and heterologous** expression

Shunjin Jia^a^, Yuchen Wu^a^, Yihui Chen^a^, Yuangui Tang^b^, Yunlu Cui^c^, Qunjian Yin^d^, Daxiong Ji^a^, Pinmei Wang^a,^* and Jinzhong Xu^a,^*

^a^Ocean College, Zhejiang University, Zhoushan, China.

^b^Shenyang Institute of Automation, Chinese Academy of Sciences, Shenyang, China

^c^College of Atmospheric Sciences, Sun Yat-sen University, Zhuhai, China

^d^Key Laboratory of Tropical Marine Ecosystem and Bioresource, Fourth Institute of Oceanography, Ministry of Natural Resources, Beihai, China

*Corresponding authors

Pinmei Wang [wangpinmei@zju.edu.cn](mailto:wangpinmei@zju.edu.cn)

Jinzhong Xu xujinzhong@zju.edu.cn

Ocean College, Zhejiang University, Zhoushan 316021, China

**Content**

[Supplementary Tables 3](#_Toc16542)

[Table S1. Strains used in this study. 4](#_Toc10289)

[Table S2. PCR primers used in this study. 5](#_Toc10115)

[Table S3. Plasmids constructed in this study. 8](#_Toc13527)

[Table S4. ^1^H and ^13^C NMR data for compounds **1** and **6** 9](#_Toc8877)

[Table S5. ^1^H and ^13^C NMR data for compounds **2** and **3** 10](#_Toc12455)

[Table S6. ^1^H and ^13^C NMR data for compounds **4** and **5** 11](#_Toc30069)

[Table S7. Putative functional homologs of OclF in *Aspergillus nidulans* LO8030.. 12](#_Toc15171)

[Supplementary Figures 13](#_Toc22495)

[Figure S1. Comparative ^13^C NMR spectra of compounds **2**, **3,** and **6**... 14](#_Toc3730)

[Figure S2. The 1D-NMR, 2D-NMR, and TOF-ESI-MS spectra of compound **1** 17](#_Toc24042)

[Figure S3. The 1D-NMR, 2D-NMR, and TOF-ESI-MS spectra of compound **2** 20](#_Toc19259)

[Figure S4. The 1D-NMR, 2D-NMR, and TOF-ESI-MS spectra of compound **3** 23](#_Toc4136)

[Figure S5. The 1D-NMR, 2D-NMR, and TOF-ESI-MS spectra of compound **4** 27](#_Toc32244)

[Figure S6. The 1D-NMR, 2D-NMR, and TOF-ESI-MS spectra of compound **5** 30](#_Toc5447)

[Supplementary References 31](#_Toc10711)

# Supplementary Tables

**Table S1**. Strains used in this study.

**Table S2.** PCR primers used in this study.

**Table S3**. Plasmids constructed in this study.

**Table S4** ^1^H and ^13^C NMR data for compounds **1** and **6**.

**Table S5.** ^1^H and ^13^C NMR data for compounds **2** and **3**.

**Table S6.** ^1^H and ^13^C NMR data for compounds **4** and **5**.

**Table S7**. Putative functional homologs of OclF in *Aspergillus nidulans* LO8030.

# Table S1. Strains used in this study.

| **Strain** | **Description** | **Purpose** | **Resource** |
| --- | --- | --- | --- |
| *Escherichia coli* DH5α | / | Plasmid cloning | In the lab |
| *Saccharomyces cerevisiae* BJ5464 | MATalpha*, ura*3-52, *trp*1, *leu2*-Δ1, *his*3-Δ200, *pep*4*::HIS*3*, prb*1*-*Δ1.6R*,*  *can*1*,* GAL | Plasmid construction | Yin et al. (2013) |
| *Aspergillus fumigatus* Af 293 | Wild type | To obtain *pyr* BGC genes | Osherov et al. (2001) |
| *Aspergillus nidulans* LO8030 | *pyr*G89, *pyro*A4, *ribo*B2, KO-DELETE :ST:::*pyr*G (Afu) integration(native); KO-DELETE :emericellamide:::*pyr*G (Afu) integration(native); KO-DELETE :asperfuranone:::*pyr*G (Afu) integration(native); KO-DELETE :monodictyphenone::*pyr*G (Afu) integration(native); KO-DELETE :terrequinone::*pyr*G (Afu) integration(native); KO-DELETE :austinol::*pyr*G (Afu) integration(native); KO-DELETE :F9775A/B::*pyr*G (Afu) integration(native);KO-DELETE :asperthecin::*pyr*G (Afu) integration(native) | Chassis strain | Chiang et al. (2016) |
| SSJ1 | *pyro*A4, *ribo*B2, pSJ1 | 1st transformants | This study |
| SSJ9 | *pyro*A4, pSJ1, Δ*yA*::(*amyBp-pyr4*-*gpdAp-pyr*5-*gpdAp-pyr6*-*AfriboB*) | 2nd transformants | This study |
| SSJ10 | pSJ1, Δ*yA*::(*amyBp-pyr4*-*gpdAp-pyr*5-*gpdAp-pyr6*-*AfriboB*),  Δ*wA*::(*amyBp-pyr3*-*gpdAp-pyr9*-*pyroA*), | 3rd transformants | This study |
| SSJ12 | pSJ1, Δ*yA*::(*amyBp-pyr4*-*gpdAp-pyr*5-*gpdAp-pyr6*-*AfriboB*),  Δ*wA*::(*amyBp-pyr3*-*gpdAp-pyr3*-*pyroA*), | 3rd transformants | This study |
| SSJ13 | pSJ1, Δ*yA*::(*amyBp-pyr4*-*gpdAp-pyr*5-*gpdAp-pyr6*-*AfriboB*),  Δ*wA*::(*amyBp-pyr9*-*gpdAp-pyr9*-*pyroA*), | 3rd transformants | This study |

# Table S2. PCR primers used in this study.

| **Name** | **Sequence (5’-3’)** | **Target fragment** |
| --- | --- | --- |
| *pyr1*-F | AATGGAGCCCCACGGCGA | *pyr1* for pSJ1 |
| *pyr1*-R | GTCGGCGTCGGGGATCCCTATGCAGGCACAGAGACACC |  |
| *pyr2-1*-F | ATGAAAGAAAATGAGCCTG | *pyr2-1* for pSJ1 |
| *pyr2-1*-R | CGTTTGTAGACAGTAGCTC |  |
| *pyr2-2*-F | AGTGATCACTAAGGAGCAC | *pyr2-2* for pSJ1 |
| *pyr2-2*-R | CCCTACACTAAATTACTAC |  |
| pSJ1-*AppyrG-AMA1*-F | TACCCAGAATGCACAGGTACACTTGTTTAGAGGTAATCCTTCTTAGCTTATACGAACAG | *pyrG*-*AMA1* for pSJ1 |
| pSJ1-*AppyrG-AMA1*-R | CAAGATTACCTATGCAATAGATGC |  |
| pSJ1-AscI*-amyBp*-F | GTATGTATTTCGGTTCCTGGAG | backbone and *amyBp* for pSJ1 |
| pSJ1-AscI*-amyBp*-R | CGACAACCTGTGCCGATAATCGCAACAGGCTCATTTTCTTTCATCGGAGCTTGCTGTGG |  |
| pSJ1-*pyr2*/*trpCt*-F | CTAGTAGTAATTTAGTGTAGGGATCCCCGACGCCGACCAAC | *pyr2*/*trpCt* for pSJ1 |
| pSJ1-*pyr2/trpCt*-R | ATTAAATGGTACTCGAGTCCAAAGAAGGATTACCTCTAAAC |  |
| pSJ1-*pyr1/trpCt*-F | GGATCCCCGACGCCGACCAACA | *pyr1/trpCt* for pSJ1 |
| pSJ1-*pyr1/trpCt*-R | AAGAAGGATTACCTCTAAACAAGTGTACC |  |
| pSJ1-*gpdAp*-F | TGGACTCGAGTACCAT | *gpdAp* for pSJ1 |
| pSJ1-*gpdAp*-R | CTAAAAGCAAAGCTGACCAGATCCGTCTCGCCGTGGGGCTCCATTGTGATGTCTGCTCA |  |
| *pyr4*-F | ATGGACGGGTGGTCAG | *pyr4* for pSJ9 |
| *pyr4*-R | TCATAGAGCTTTCTTCCGC |  |
| *pyr5*-F | ATGAGAGTCCTCATTATTGG | *pyr5* for pSJ9 |
| *pyr5*-R | CTATACAGTCAACGTGGAATG |  |
| *pyr6*-F | ATGGCCACCGCTCA | *pyr6* for pSJ9 |
| *pyr6*-R | CTAGAACATATCCAGAGACC |  |
| pSJ9-*yA*up-F | GATGGAGATTGGCGCC | *yA-*up for pSJ9 |
| pSJ9-*yA*up-R | TCGGCTTGATGTTGTTCGC |  |
| pSJ9-*yA*down-F | CTTGTTGGACGGGGTTGA | *yA-*down for pSJ9 |
| pSJ9-*yA*down-R | TCGGCGTTGAGCGCGTCAT |  |
| pSJ9-*AfriboB*-F | GGTCTCTGGATATGTTCTAGGCGGACTGAGTTATGGATG | *AfriboB* for pSJ9 |
| pSJ9-*AfriboB*-R | TCACCTAAGAATCCCAAACATCAACCCCGTCCAACAAGGGCTGCCACTCAACGCCATTG |  |
| pSJ9-AscI-F | TCGTATACGCCAGGGAGGGCAATGACGCGCTCAACGCCGATGCTTTACGGAAGACAATG | backbone for pSJ9 |
| pSJ9-AscI-R | TGGTGGCACAACAGATAAAGAACGGCGCCAATCTCCATCGGGTGCCTAATGAGTGAGCT |  |
| pSJ9-*amyBp*-F | AGCGAACAACATCAAGCCGACCATCATGGTGTTTTGATC | *amyBp* for pSJ9 |
| pSJ9-*amyBp*-R | CTGTACTGGGGAGGTGCCGATGAGAGATCTGACCACCCGTCCATCGGAGCTTGCTGTGG |  |
| pSJ9-*gpdAp*/*pyr5*-F | AGCGGAAGAAAGCTCTATGATGGACTCGAGTACCAT | *gpdAp*/*pyr5* for pSJ9 |
| pSJ9-*gpdAp*/*pyr5*-R | GAGACCGGCGATGGATCCTCCAATAATGAGGACTCTCATTGTGATGTCTGCTCAAGCGG |  |
| pSJ9-*gpdAp*/*pyr6*-F | ATTCCACGTTGACTGTATAGTGGACTCGAGTACCAT | *gpdAp*/*pyr6* for pSJ9 |
| pSJ9-*gpdAp*/*pyr6*-R | CTGAGCGGTGGCCATTGTGATGTCTGCTCAAGCGG |  |
| pSJ10-*pyr3*-F | ATGGTATCTAGCTTCTCG | *pyr3* for pSJ10 |
| pSJ10-*pyr3*-R | CTTGCATTCCACAGACTG |  |
| pSJ10-*pyr9*-F | ATGATCAGAGTTGAAGATGC | *pyr9* for pSJ10 |
| pSJ10-*pyr9*-R | GCTGTATGAATACTCTGACCAG |  |
| pSJ10-*wA*up-F | CTCTGGAACAGTCTCG | *wA-*up for pSJ10 |
| pSJ10-*wA*up-R | GATCAGGAGAAGGAGAGTCAAG |  |
| pSJ10-*wA*down-F | GGCAAAGGAGCTTGCTA | *wA-*down for pSJ10 |
| pSJ10-*wA*down-R | CGACTTCGGGAATTGAAC |  |
| pSJ10-*amyBp*-F | AATTGCCTGATCAGCGGACTTGACTCTCCTTCTCCTGATCCCATCATGGTGTTTTGATC | *amyBp* for pSJ10 |
| pSJ10-*amyBp*-R | TCCATGGTGTCTCCAAGTCGCTTGGACGAGAAGCTAGATACCATCGGAGCTTGCTGTGG |  |
| pSJ10-*gpdAp*-F | GGGTGATAAGAGGGAATCTACGGACAGTCTGTGGAATGCAAGTGGACTCGAGTACCATT | *gpdAp* for pSJ10 |
| pSJ10-*gpdAp*-R | ACCCATACCGTCCCGATGGACGCATCTTCAACTCTGATCATTGTGATGTCTGCTCAAGC |  |
| pSJ10-*pyroA*-F | ACTCTATTTTGATGCGATGCTGGTCAGAGTATTCATACAGCGCATCCACATGATCGACA | *pyroA* for pSJ10 |
| pSJ10-*pyroA*-R | ACCCCCAACGCATTCTTCATGAAAGTAGCAAGCTCCTTTGCCTGCAGAATTCGCCCTTA |  |
| pSJ10-AscI-F | TCATCGTTTTGGATGTTCAATTCCCGAAGTCGCAAAATCTGCAGGATCCTCTAGAGTGC | backbone for pSJ10 |
| pSJ10-AscI-R | GGTCAAATGCACGAGCAAGACGGCGAGACTGTTCCAGAGGGGTGCCTAATGAGTGAGCT |  |
| pSJ12-*pyr3*-F | GCAGCTTGACTAACAGCTACCCCGCTTGAGCAGACATCACAATGGTATCTAGCTTCTCG | *pyr3* for pSJ12 |
| pSJ12-*pyr3*-R* | CTTGCATTCCACAGACTG |  |
| pSJ12-*pyroA*-*wA*down-AscI-F | GGTGATAAGAGGGAATCTACGGACAGTCTGTGGAATGCAAGGCATCCACATGATCGACA | *pyro*-*wA*down and backbone for pSJ12 |
| pSJ12-*pyroA*-*wA*down-AscI-R* | GGTCAAATGCACGAGCAAGACGGCGAGACTGTTCCAGAGGGGTGCCTAATGAGTGAGCT |  |
| pSJ12-*wA*up-*amyBp*-*pyr3-gpdAp-*F* | CTCTGGAACAGTCTCG | *wA*up-*amyBp*-*pyr3-gpdAp* for pSJ12 |
| pSJ12-*wA*up-*amyBp*-*pyr3-gpdAp-*R* | TGTGATGTCTGCTCAAGCGG |  |
| pSJ13-*pyr9*-F | TCTCCCTTCTCTGAACAATAAACCCCACAGCAAGCTCCGATGATCAGAGTTGAAGATGC | *pyr9* for pSJ13 |
| pSJ13-*pyr9*-R* | GCTGTATGAATACTCTGACCAG |  |
| pSJ13-*gpdAp-pyr9-pyroA*-*wA*down-F | GACTCTATTTTGATGCGATGCTGGTCAGAGTATTCATACAGCTGGACTCGAGTACCATT | *gpdAp-pyr9-pyroA*-*wA*down for pSJ13 |
| pSJ13-*gpdAp-pyr9-pyroA*-*wA*down-R* | CGACTTCGGGAATTGAAC |  |
| pSJ13-AscI-*wA*up-*amyBp-*F* | TCATCGTTTTGGATGTTCAATTCCCGAAGTCGCAAAATCTGCAGGATCCTCTAGAGTGC | backbone and *wA*up-*amyBp* for pSJ13 |
| pSJ13-AscI-*wA*up-*amyBp-*R* | CGGAGCTTGCTGTGGGGTTT |  |

*Reused primers

# Table S3. Plasmids constructed in this study.

| **Name** | **Description** | **Purpose** |
| --- | --- | --- |
| pSJ1 | *amyBp-pyr*2-*trp*C*t*-*gpdAp-pyr*1-*trp*C*t*-*AppyrG-AMA1* | Heterologous expression of *pyr*1 and *pyr*2 |
| pSJ9 | *amyBp-pyr4*-*gpdAp-pyr*5-*gpdAp-pyr6*-*AfriboB* | Contain cassette for 2^nd^ transformation |
| pSJ10 | *amyBp-pyr3*-*gpdAp-pyr9*-*pyroA, AMA1* | Contain cassette for 3^nd^ transformation |
| pSJ12 | *amyBp-pyr3*-*gpdAp-pyr3*-*pyroA, AMA1* | Contain cassette for 3^nd^ transformation |
| pSJ13 | *amyBp-pyr9*-*gpdAp-pyr9*-*pyroA, AMA1* | Contain cassette for 3^nd^ transformation |

The structures of compounds **1****–6**.

# Table S4. ^1^H and ^13^C NMR data for compounds 1 and 6.

| **Position** | **Compound 1 (in CDCl_3_)** | | **Deacetyl-PPE (6) (in CDCl_3_)** | |
| --- | --- | --- | --- | --- |
|  | *δ*_H_ (*J* in Hz) | *δ*_C_ | *δ*_H_ (*J* in Hz) | *δ*_C_ |
| 1 | - | 216.0 | 3.25 (dd, *J* = 11.5, 4.5) | 78.4 |
| 2 | 2.48 (m)  2.61 (m) | 33.7 | 1.58 - 1.75 (m)  1.58 - 1.75 (m) | 27.1 |
| 3 | 1.55 (m)  2.07 (m) | 37.9 | 1.12 (dt, *J* = 13.5. 4.0)  1.77 - 1.85 (m) | 37.4 |
| 4 | - | 36.7 | - | 36.8 |
| 5 | 1.60 (m) | 50.9 | 1.50 (dd, *J* = 13.0, 4.5) | 51.5 |
| 6 | - | 80.7 | - | 81.0 |
| 7 | 1.73 (m)  2.19 (m) | 39.7 | 1.58 - 1.75 (m)  2.14 (dt, *J* = 13.0, 3.0) | 40.3 |
| 8 | 1.56 (m)  1.77 (m) | 20.5 | 1.44 (m)  1.77 - 1.85 (m) | 19.3 |
| 9 | 1.58 (m) | 54.7 | 1.00 (dd, *J* = 12.5, 1.5) | 54.9 |
| 10 | - | 47.3 | - | 38.8 |
| 11 | 1.14 (s) | 26.6 | 0.81 (s) | 15.5 |
| 12 | 1.06 (s) | 17.5 | 0.91 (s) | 15.1 |
| 13 | 2.31 (dd, *J* = 17.0, 12.9)  2.57 (m) | 17.5 | 2.24 (dd, *J* = 17.5, 13.0)  2.52 (dd, *J* = 17.5, 4.5) | 17.2 |
| 14 | 1.33 (s) | 20.5 | 1.27 (s) | 20.7 |
| 15 | 1.08 (s) | 21.3 | 1.03 (s) | 28.1 |
| 2’ | - | 163.8 | - | 164.0 |
| 3’ | - | 100.4 | - | 100.3 |
| 4’ | - | 162.7 | - | 162.8 |
| 5’ | 6.46 (s) | 99.6 | 6.42 (s) | 99.4 |
| 6’ | - | 155.4 | - | 155.5 |
| 2” | 9.01 (br.s) | 146.0 | 8.98 (br.s) | 146.6 |
| 3” | - | 127.9 | - | 127.6 |
| 4” | 8.17 (dt, *J* = 8.1,2.0) | 133.5 | 8.10 (m) | 132.8 |
| 5” | 7.45 (dd, *J* = 8.2,4.8) | 123.9 | 7.38 (dd, *J* = 8.0, 5.0) | 123.6 |
| 6” | 8.67 (d, *J* = 4.0) | 150.3 | 8.64 (br d, *J* = 5.0) | 151.0 |

*^1^H NMR: 600 MHz, ^13^C NMR: 150 MHz.

# Table S5. ^1^H and ^13^C NMR data for compounds 2 and 3.

| **Position** | **Compound 2 (in CDCl_3_)** | | **Compound 3 (in DMSO-*d*_6_)** | |
| --- | --- | --- | --- | --- |
|  | *δ*_H_ (*J* in Hz) | *δ*_C_ | *δ*_H_ (*J* in Hz) | *δ*_C_ |
| 1 | - | 216.0 | 3.03 (dd, *J* = 11.4, 4.7) | 76.6 |
| 2 | 2.46 (m)  2.77 (m) | 33.8 | 1.59 (m)  1.59 (m) | 26.8 |
| 3 | 1.67 (m)  2.41 (m) | 37.5 | 1.21 (td, *J* = 13.0, 4.1)  1.98 (dt, *J* = 12.7, 3.4) | 36.3 |
| 4 | - | 38.0 | - | 37.7 |
| 5 | 1.56 (d, *J* = 4.2) | 55.6 | 1.35 (d, *J* = 3.4) | 55.3 |
| 6 | - | 82.0 | - | 81.8 |
| 7 | 1.74 (m)  2.18 (m) | 41.0 | 1.64 (m)  2.03 (m) | 41.6 |
| 8 | 1.74 (m) | 20.7 | 1.52 (m)  1.70 (m) | 19.3 |
|  | 1.49 (m) | 55.9 | 0.93 (br.s) | 54.7 |
| 10 | - | 47.4 | - | 38.4 |
| 11 | 1.12 (s) | 26.5 | 0.91 (s) | 28.3 |
| 12 | 1.51 (s) | 16.4 | 1.26 (s) | 16.6 |
| 13 | 5.00 (d, *J* = 4.2) | 60.4 | 4.77 (d, *J* = 3.1) | 58.2 |
| 14 | 1.70 (s) | 22.1 | 1.61 (s) | 22.3 |
| 15 | 1.10 (s) | 21.6 | 0.71 (s) | 15.9 |
| 2’ | - | 164.2 | - | 162.3 |
| 3’ | - | 103.5 | - | 103.4 |
| 4’ | - | 162.7 | - | 162.1 |
| 5’ | 6.47 (s) | 99.5 | 6.98 (s) | 99.4 |
| 6’ | - | 157.3 | - | 156.2 |
| 2” | 9.01 (d, *J* = 1.8) | 146.8 | 9.05 (d, *J* = 2.4) | 146.5 |
| 3” | - | 127.3 | - | 127.0 |
| 4” | 8.12 (dt, *J* = 8.2, 2.0) | 133.0 | 8.23 (dt, *J* = 8.2, 1.7) | 132.8 |
| 5” | 7.42 (dd, *J* = 8.1, 4.8) | 123.7 | 7.53 (dd, *J* = 8.1, 4.8) | 123.9 |
| 6” | 8.69 (dd, *J* = 4.9, 1.6) | 151.5 | 8.67 (dd, *J* = 4.7, 1.6) | 151.2 |
| 1-OH | - | - | 4.39 (s) | - |
| 13-OH | - | - | 5.25 (s) | - |

*^1^H NMR: 600 MHz, ^13^C NMR: 150 MHz.

# Table S6. ^1^H and ^13^C NMR data for compounds 4 and 5.

| **Position** | **Compound 4 (in DMSO-*d*_6_)** | | **Compound 5 (in DMSO-*d*_6_)** | |
| --- | --- | --- | --- | --- |
|  | *δ*_H_ (*J* in Hz) | *δ*_C_ | *δ*_H_ (*J* in Hz) | *δ*_C_ |
| 1 | 3.03 (m, *J* = 9.8, 5.2) | 76.3 | - | 215.3 |
| 2 | 1.59 (m)  1.59 (m) | 26.9 | 2.34 (m)  2.70 (m) | 33.5 |
| 3 | 1.17 (td, *J* = 12.9, 4.2)  1.96 (dt, *J* = 12.9, 3.5) | 36.1 | 1.63 (m)  2.20 (m) | 36.1 |
| 4 | - | 37.6 | - | 37.3 |
| 5 | 1.25 (d, *J* = 3.4) | 53.7 | 1.42 (d, *J* = 3.4) | 2 |
| 6 | - | 85.4 | - | 85.0 |
| 7 | 3.58 (m, *J* = 10.3, 5.2) | 76.5 | 3.63 (m, *J* = 10, 5.0) | 75.6 |
| 8 | 1.46 (q, *J* = 12.4)  1.74 (dd, *J* = 12.4, 5.0) | 29.1 | 1.59 (m)  1.68 (m) | 29.8 |
| 9 | 0.97 (dd, *J* = 12.4,2.0) | 51.8 | 1.60 (m) | 51.4 |
| 10 | - | 38.2 | - | 46.3 |
| 11 | 0.90 (s) | 28.3 | 1.01 (s) | 26.1 |
| 12 | 1.24 (s) | 16.6 | 1.38 (s) | 15.9 |
| 13 | 4.76 (d, *J* = 5.7) | 58.3 | 4.77 (d, *J* = 5.7) | 58.3 |
| 14 | 1.53 (s) | 15.5 | 1.57 (s) | 15.3 |
| 15 | 0.71 (s) | 15.9 | 0.98 (s) | 21.2 |
| 2’ | - | 162.4 | - | 162.4 |
| 3’ | - | 103.1 | - | 103.1 |
| 4’ | - | 162.1 | - | 162.1 |
| 5’ | 6.90 (s) | 99.4 | 6.91 (s) | 99.4 |
| 6’ | - | 156.2 | - | 156.3 |
| 2” | 9.05 (d, *J* = 2.4) | 146.5 | 9.06 (d, *J* = 2.4) | 146.5 |
| 3” | - | 127.1 | - | 127.1 |
| 4” | 8.23 (dt, *J* = 8.1, 1.6) | 132.9 | 8.23 (dt, *J* = 8.0, 2.1) | 133.0 |
| 5” | 7.53 (dd, *J* = 8.1, 4.8) | 123.9 | 7.54 (dd, *J* = 8.0, 4.8) | 124.0 |
| 6” | 8.67 (dd, *J* = 4.8, 1.6) | 151.2 | 8.68 (dd, *J* = 4.8, 2.1) | 151.3 |
| 1-OH | 4.37 (d, *J* = 5.2) | - | - | - |
| 7-OH | 4.98 (d, *J* = 5.0) | - | 5.12 (d, *J* = 5.3) | - |
| 13-OH | 5.24 (d, *J* = 5.7) | - | 5.41 (d, *J* = 5.7) | - |

*^1^H NMR: 600 MHz, ^13^C NMR: 150 MHz.

# Table S7. Putative functional homologs of OclF in *Aspergillus nidulans* LO8030.

| **Gene name** | **Accession number** | **Protein Length (aa)** | **Query Cover (%)** | **Identity (%)** | **Deduced function** |
| --- | --- | --- | --- | --- | --- |
| AN8113 | XP_681382.1 | 268 | 99 | 26.2 | Putative oxidoreductase with a predicted role in carbohydrate metabolism |
| AN1822 | XP_659426.1 | 285 | 97 | 30.7 | Has domain(s) with predicted oxidoreductase activity |
| AN9284 | XP_682553.1 | 254 | 98 | 25.3 | Ortholog of Asptu1_0059406 |

# Supplementary Figures

**Figure S1.** Comparative ^13^C NMR spectra of compounds **2**, **3,** and **6**.

**Figure S2.** The 1D-NMR, 2D-NMR, and TOF-ESI-MS spectra of compound **1**.

**Figure S3.** The 1D-NMR, 2D-NMR, and TOF-ESI-MS spectra of compound **2**.

**Figure S4.** The 1D-NMR, 2D-NMR, and TOF-ESI-MS spectra of compound **3**.

**Figure S5.** The 1D-NMR, 2D-NMR, and TOF-ESI-MS spectra of compound **4**.

**Figure S6.** The 1D-NMR, 2D-NMR, and TOF-ESI-MS spectra of compound **5**.


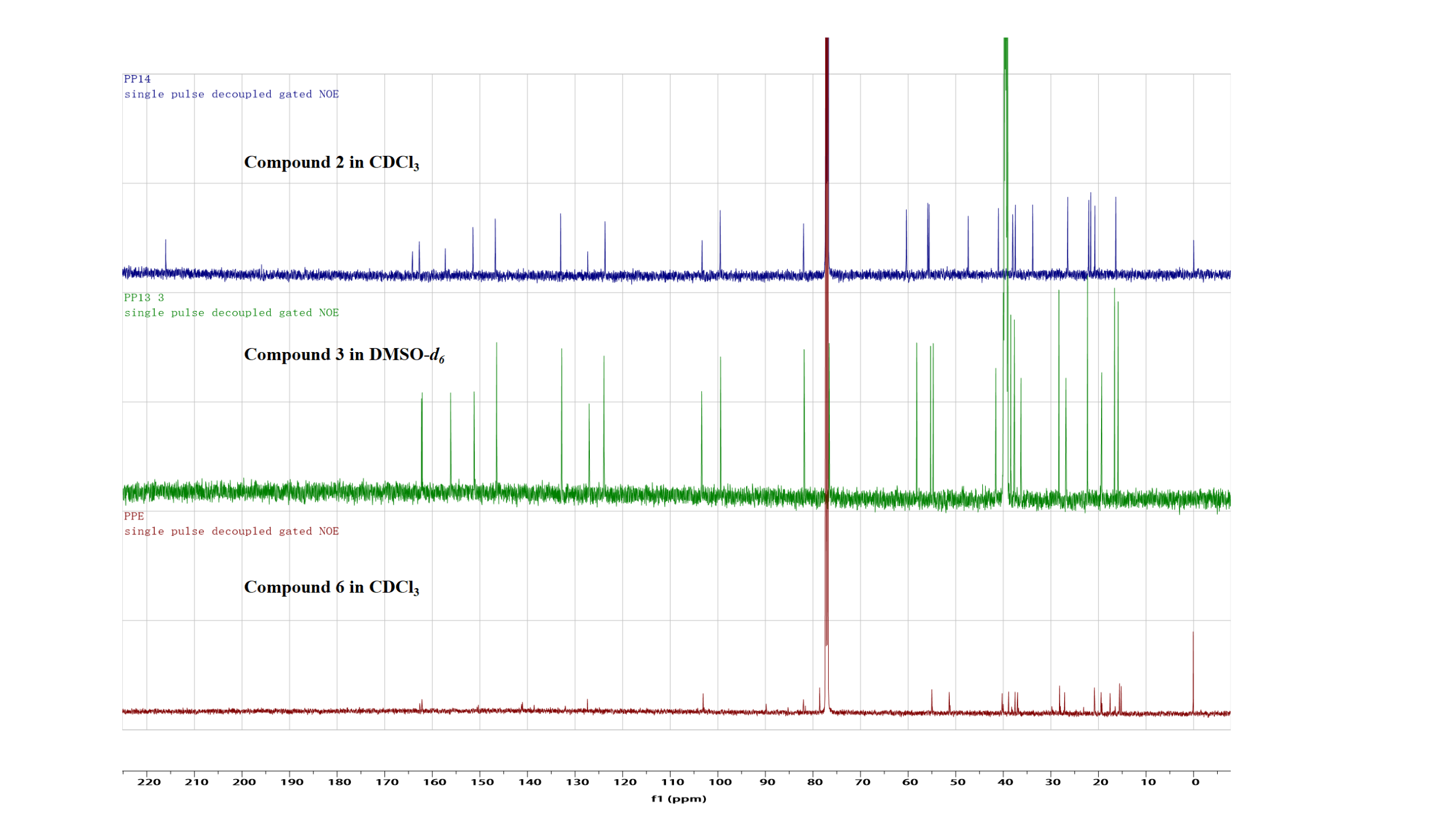


# Figure S1. Comparative ^13^C NMR spectra of compounds 2, 3, and 6.


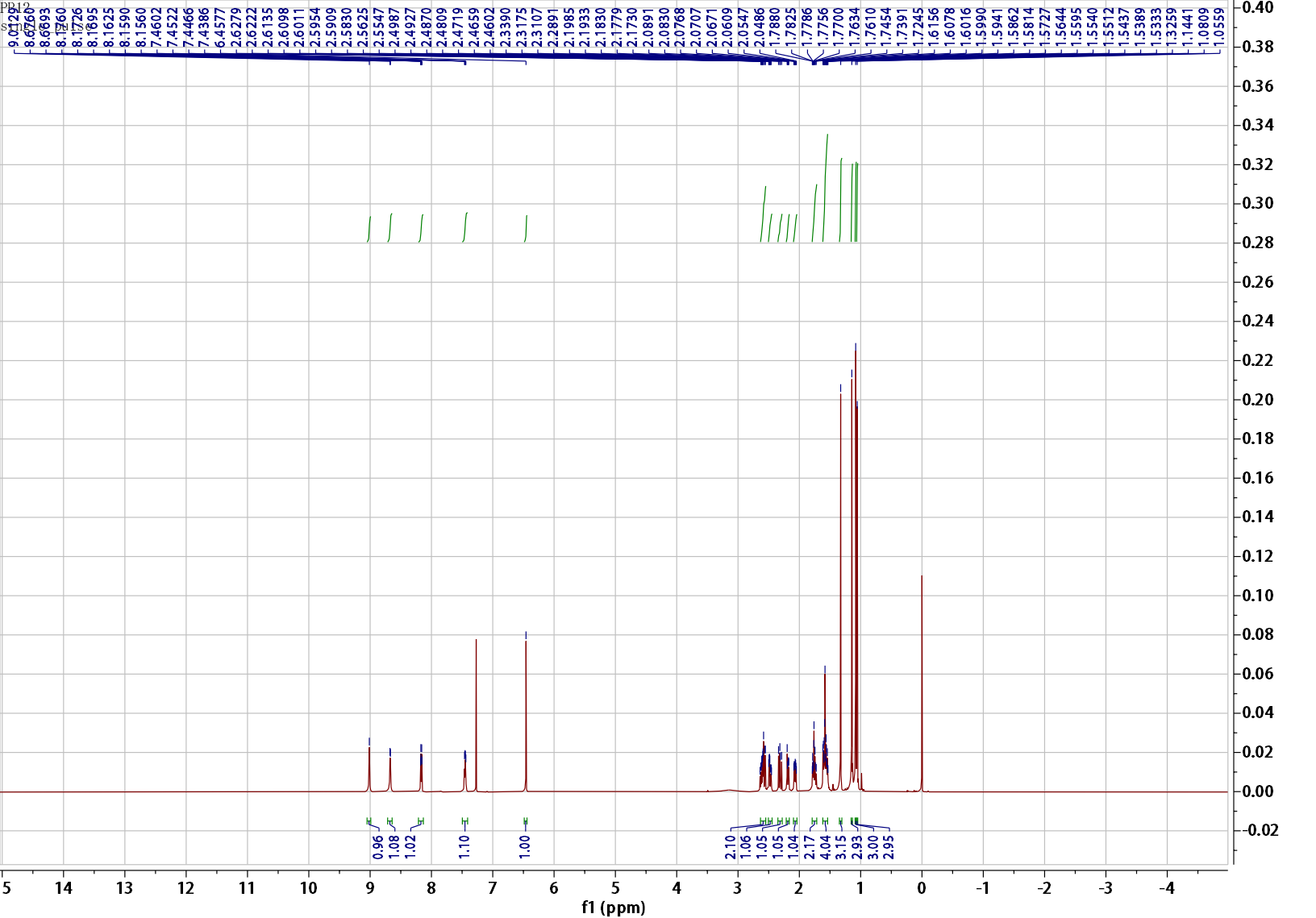


The ^1^H NMR spectrum of compound **1** in CDCl_3_


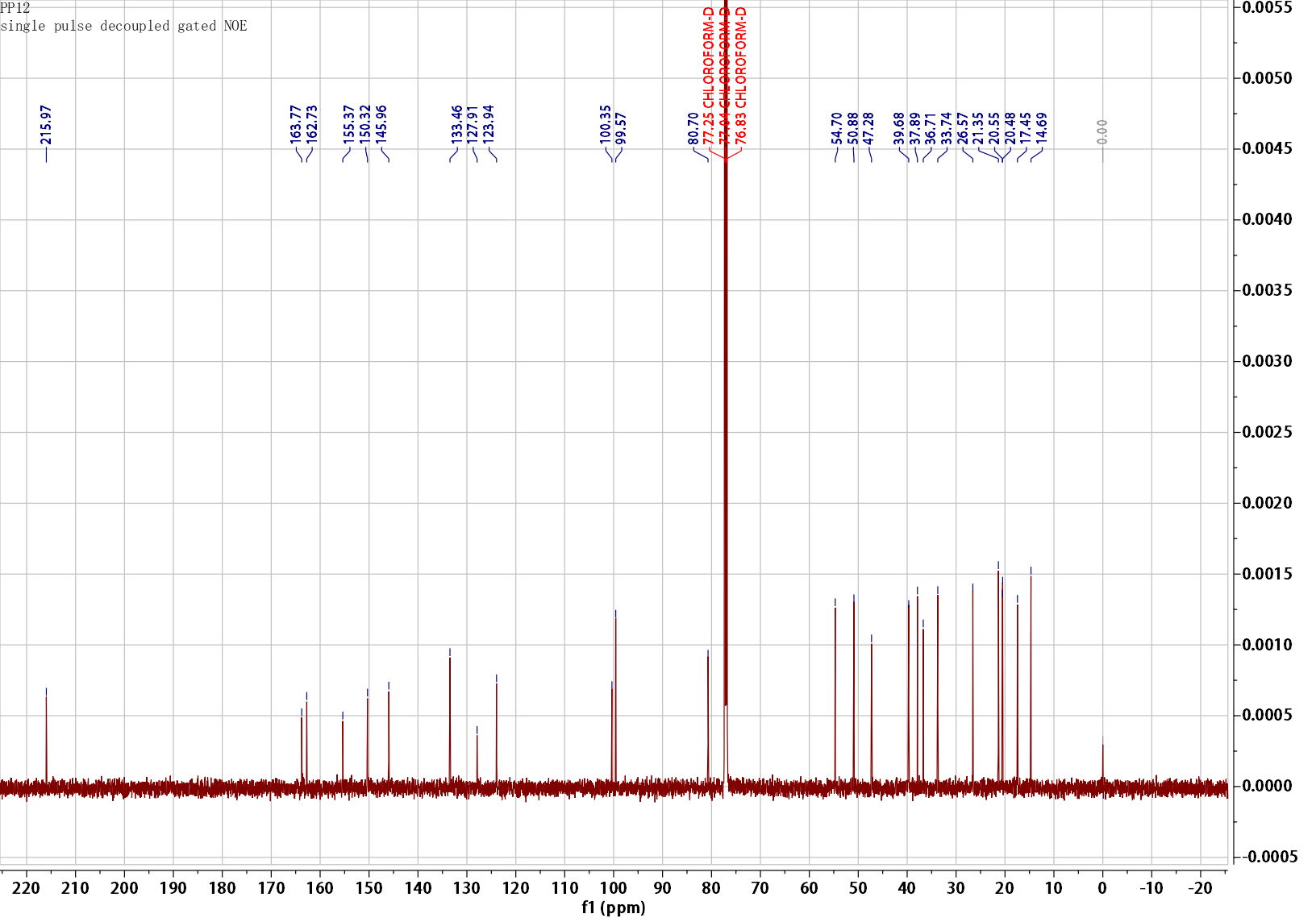


The ^13^C NMR spectrum of compound **1** in CDCl_3_


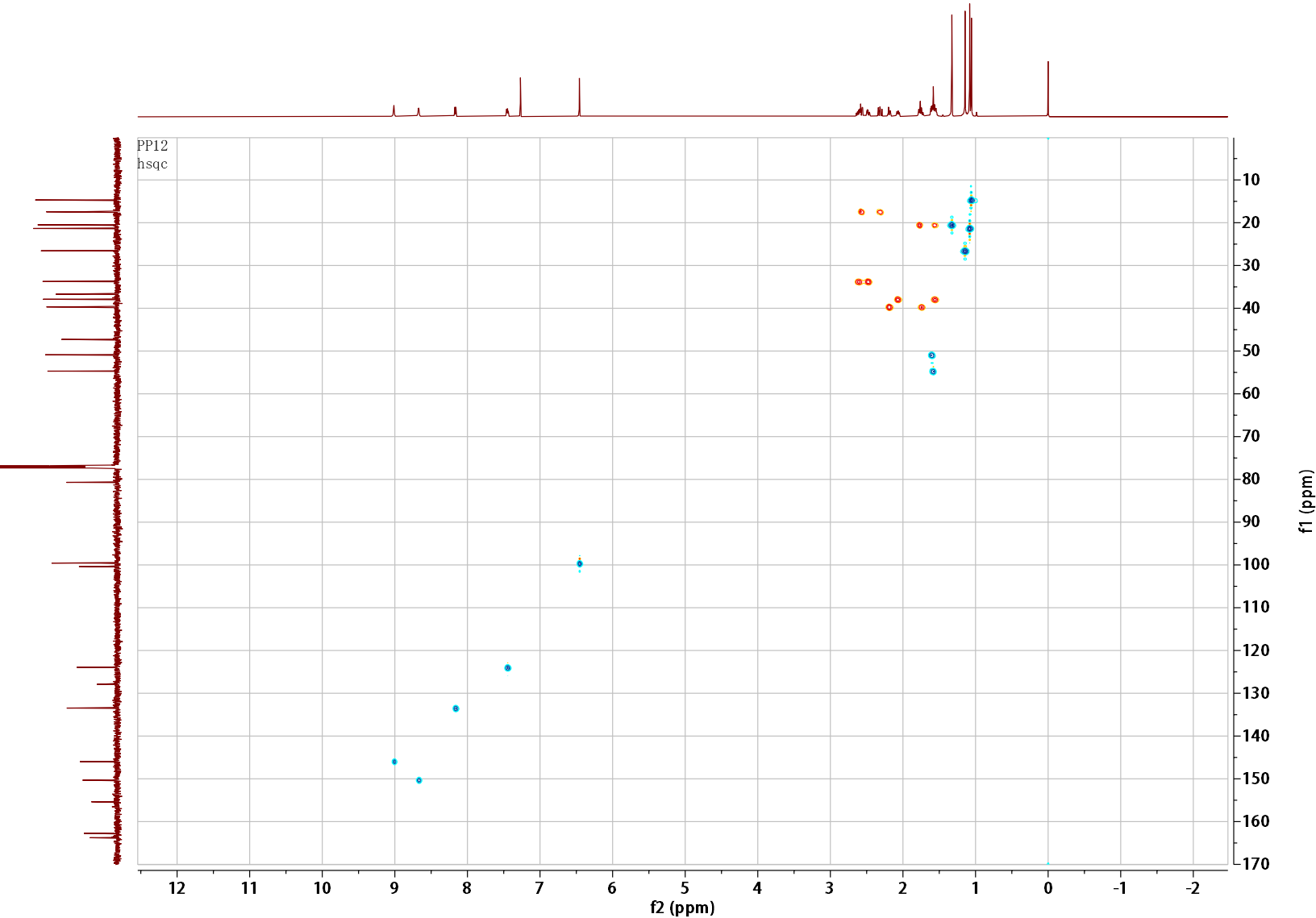


The HSQC spectrum of compound **1** in CDCl_3_


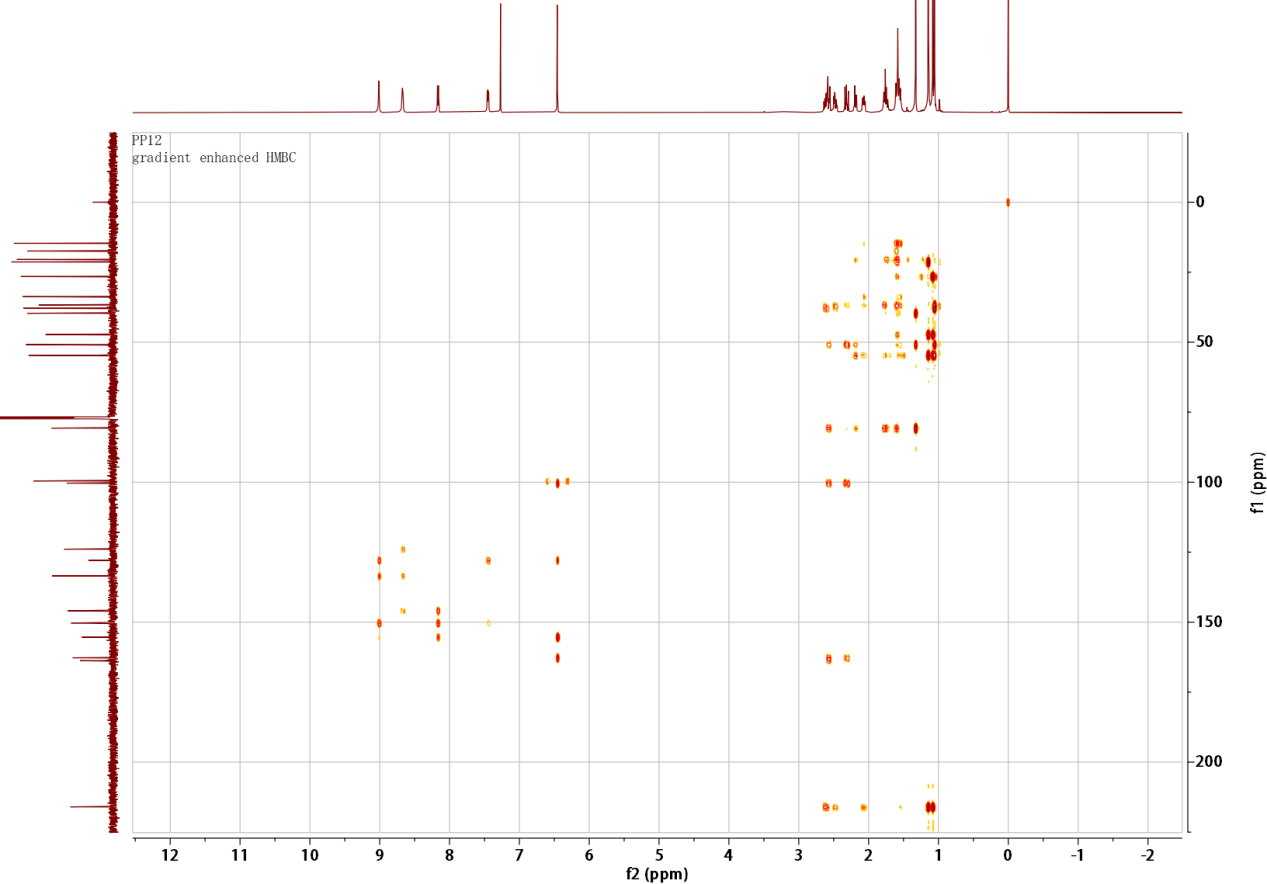


The HMBC spectrum of compound **1** in CDCl_3_


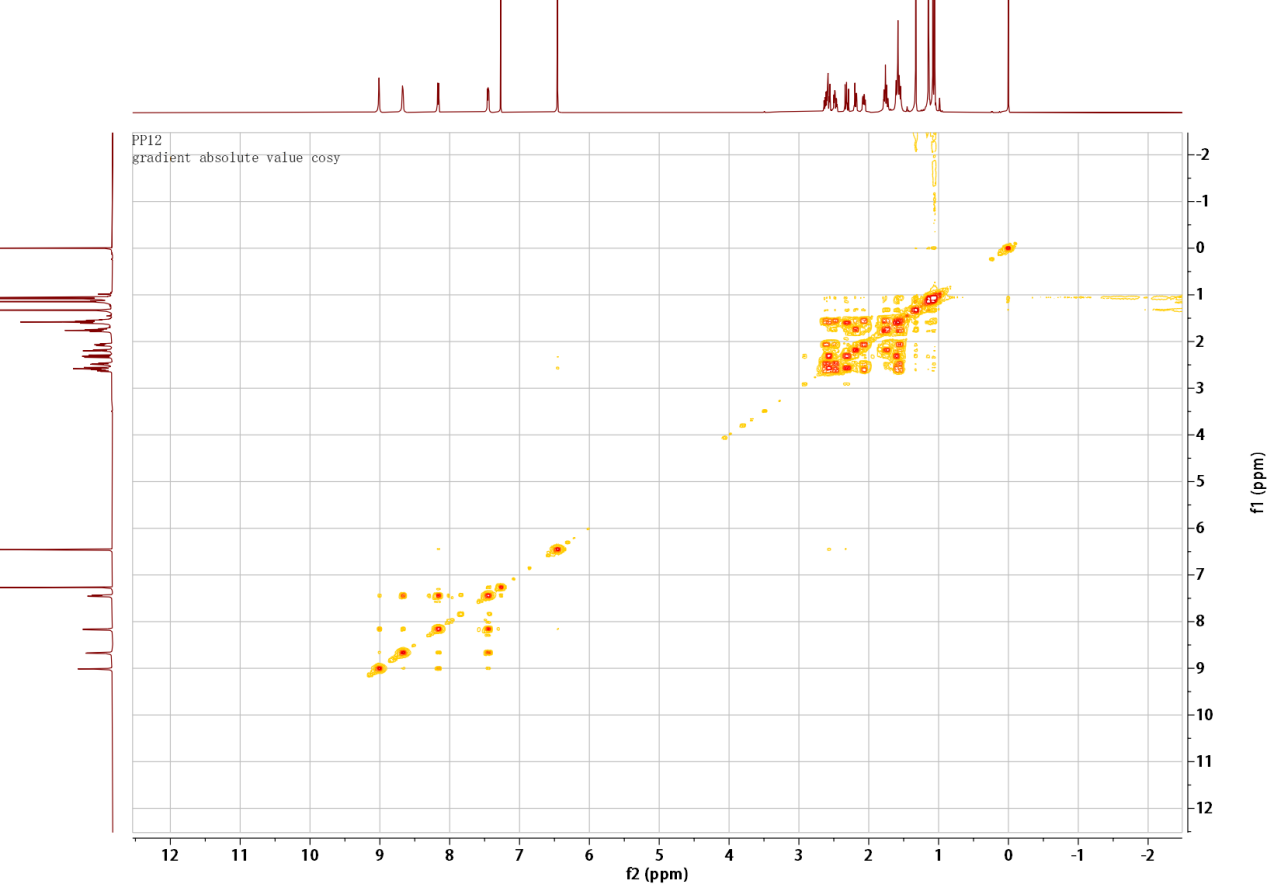


The ^1^H-^1^H COSY spectrum of compound **1** in CDCl_3_


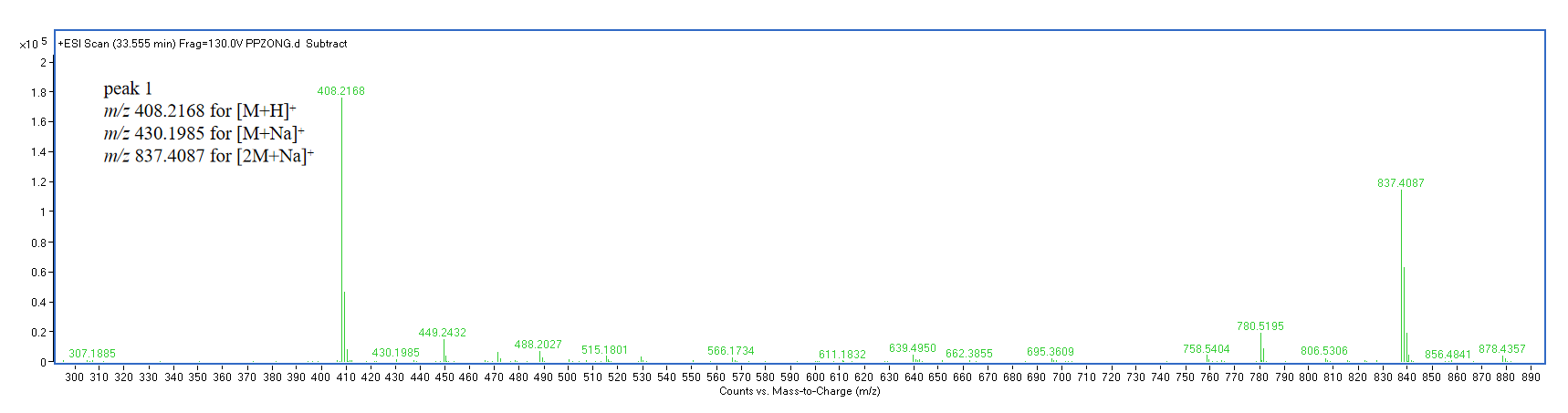


The TOF-ESI-MS spectrum of compound **1**

# Figure S2. The 1D-NMR, 2D-NMR, and TOF-ESI-MS spectra of compound 1.


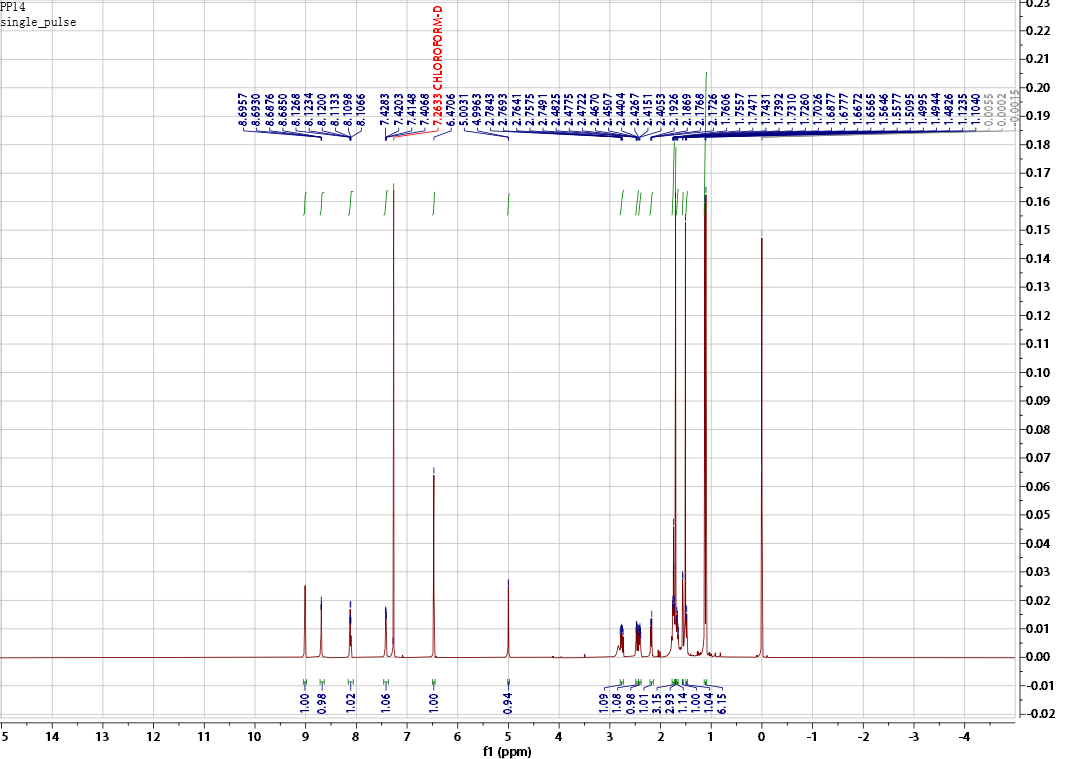


The ^1^H NMR spectrum of compound **2** in CDCl_3_


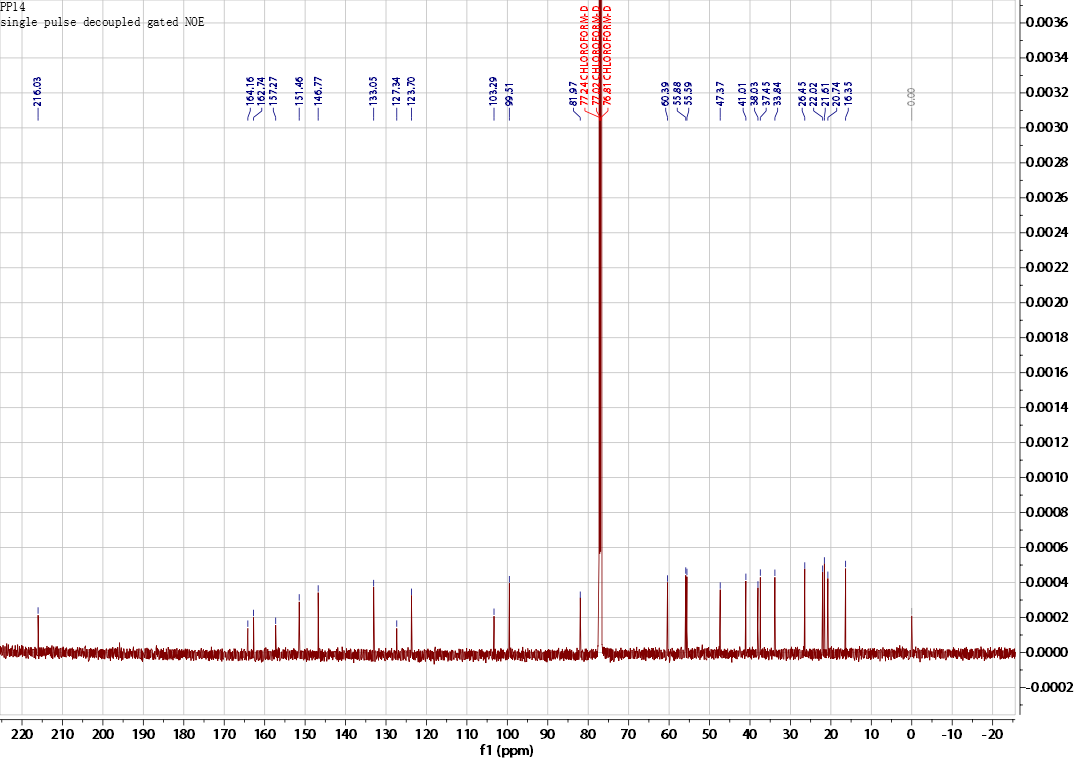


The ^13^C NMR spectrum of compound **2** in CDCl_3_


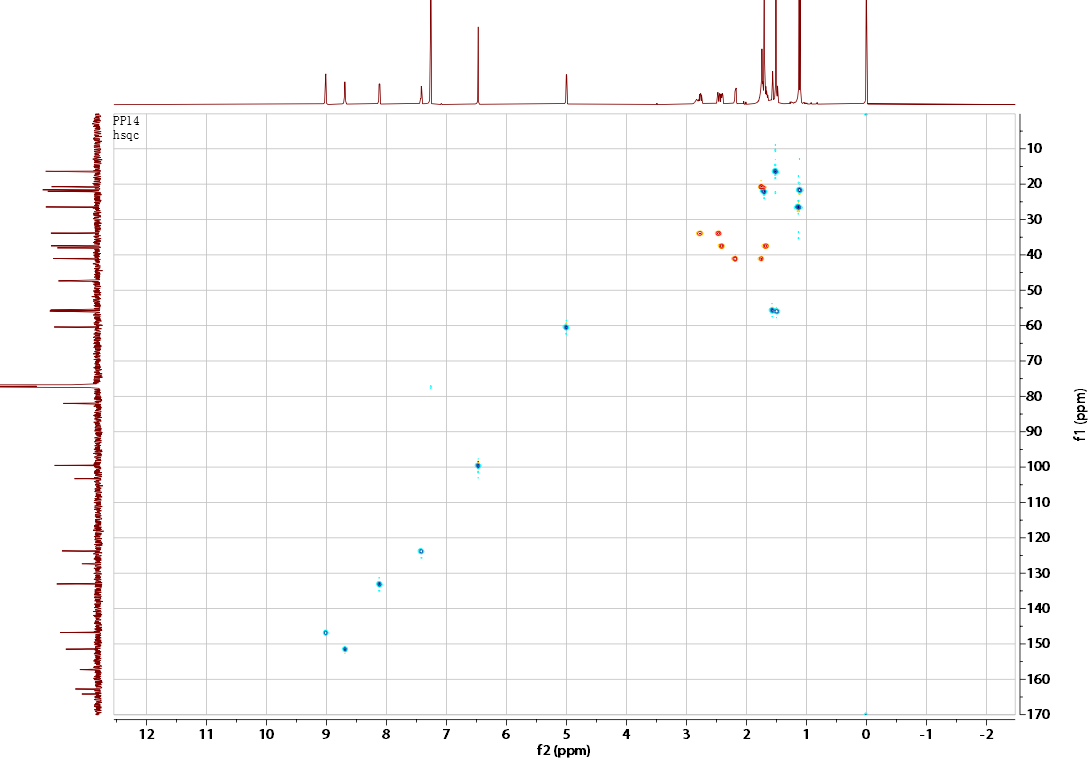


The HSQC spectrum of compound **2** in CDCl_3_


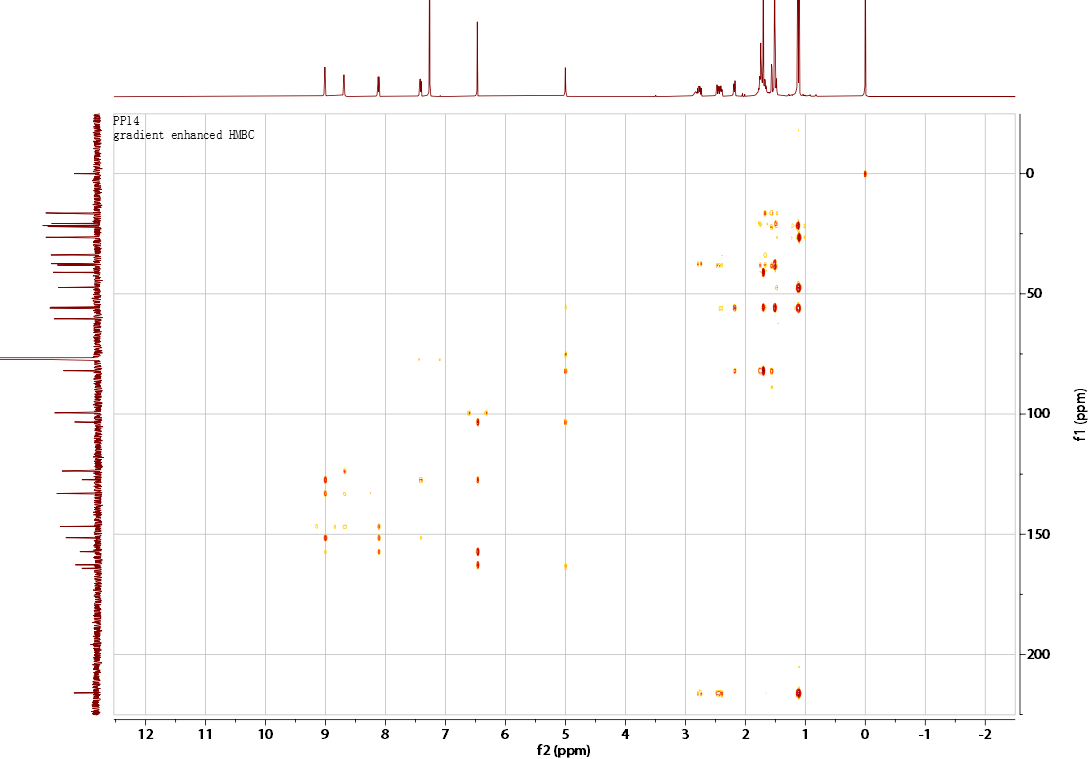


The HMBC spectrum of compound 2 in CDCl_3_


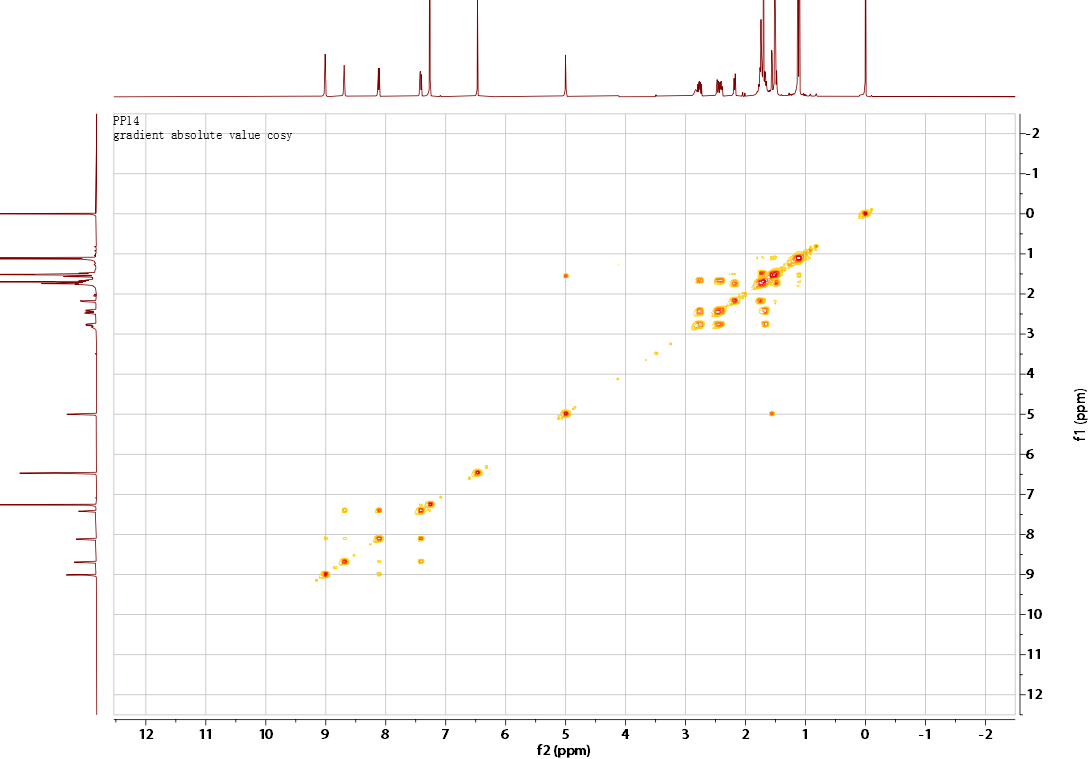


The ^1^H-^1^H COSY spectrum of compound **2** in CDCl_3_


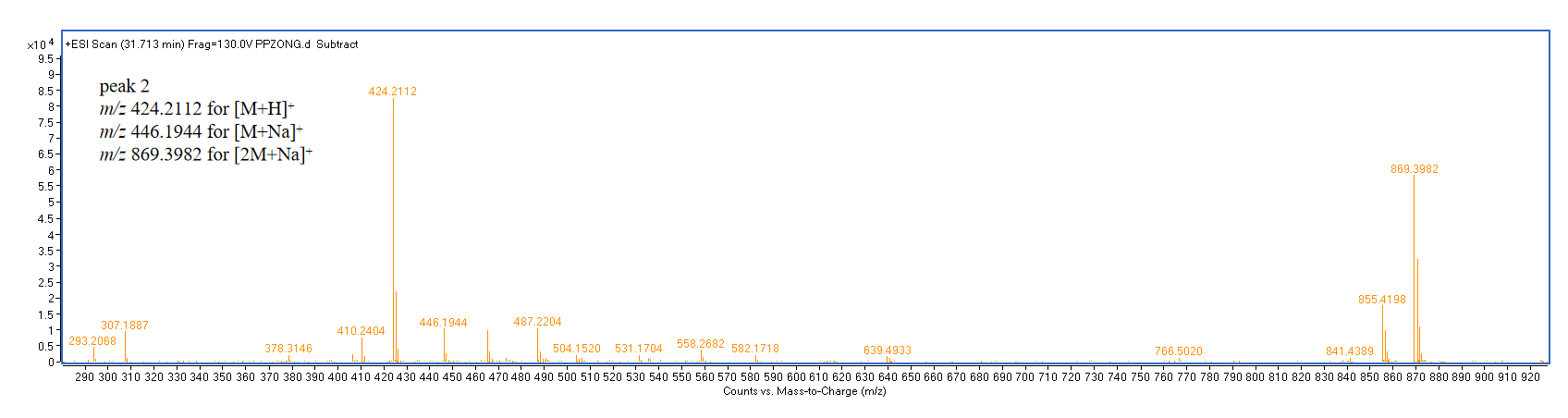


The TOF-ESI-MS spectrum of compound **2**

# Figure S3. The 1D-NMR, 2D-NMR, and TOF-ESI-MS spectra of compound 2.


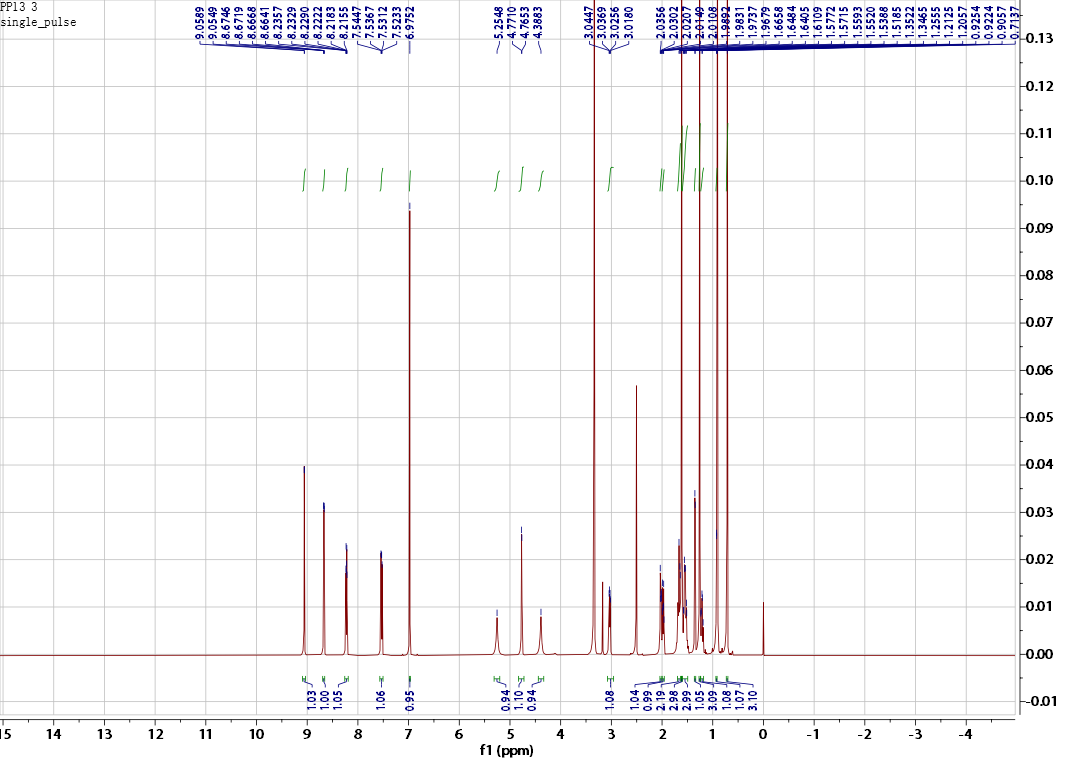


The ^1^H NMR spectrum of compound **3** in DMSO-*d*_6_


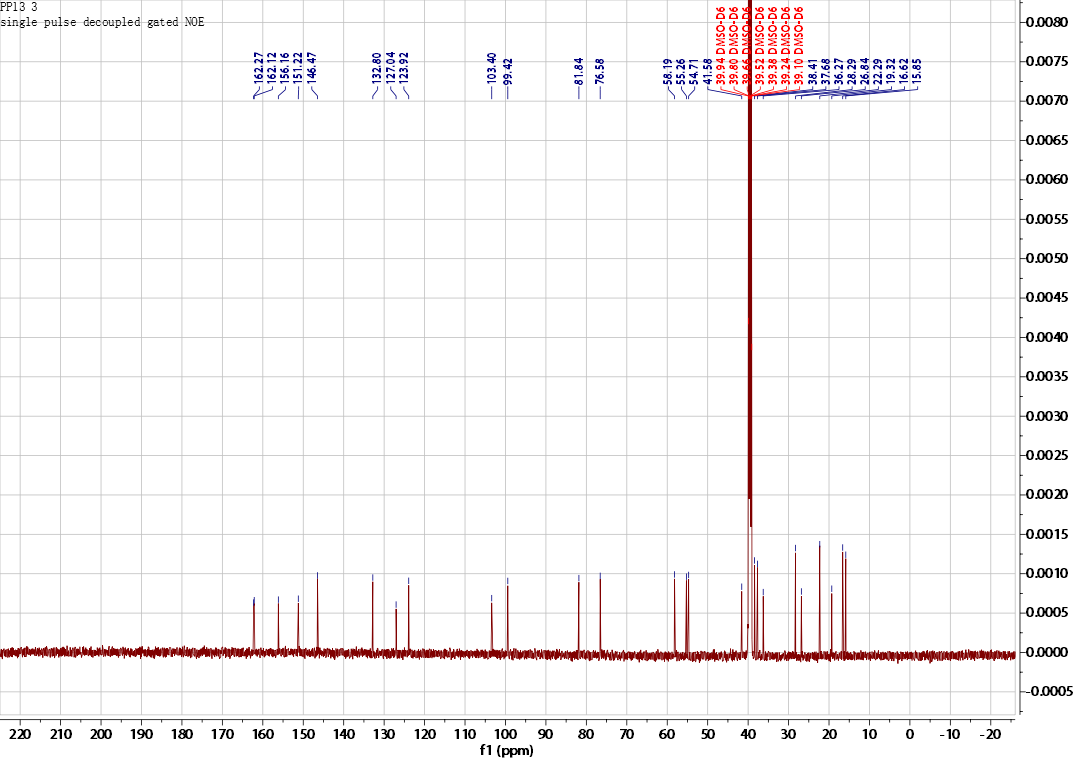


The ^13^C NMR spectrum of compound **3** in DMSO-*d*6


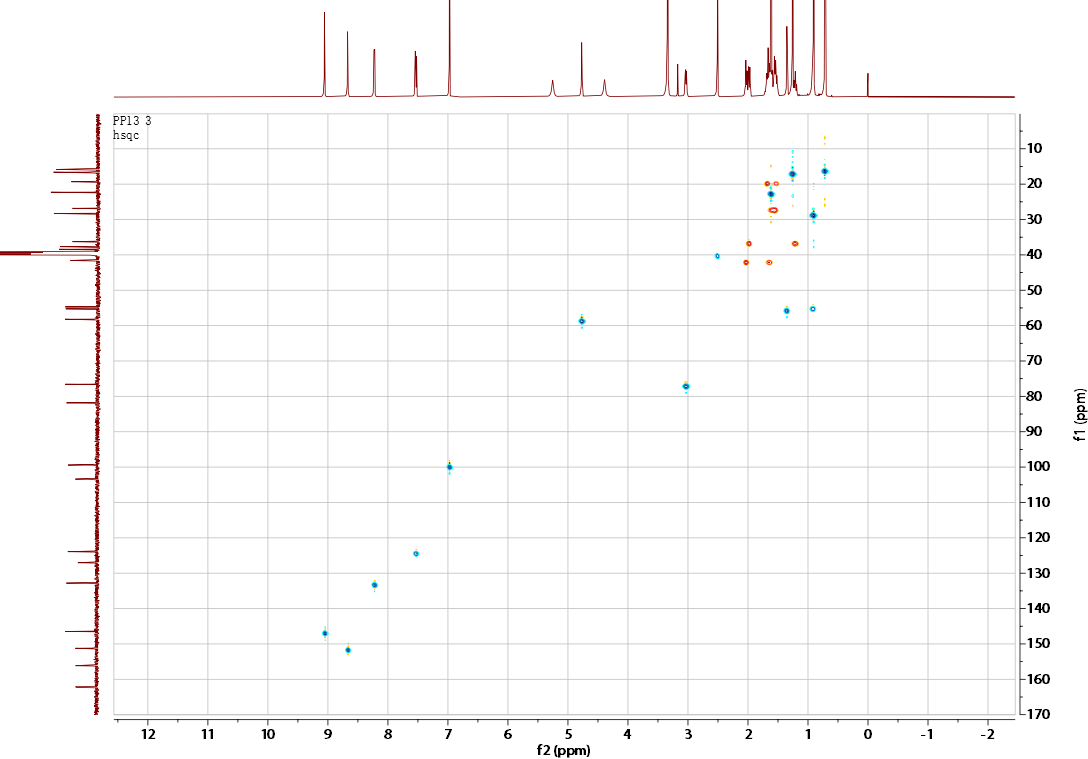


The HSQC spectrum of compound **3** in DMSO-*d*_6_


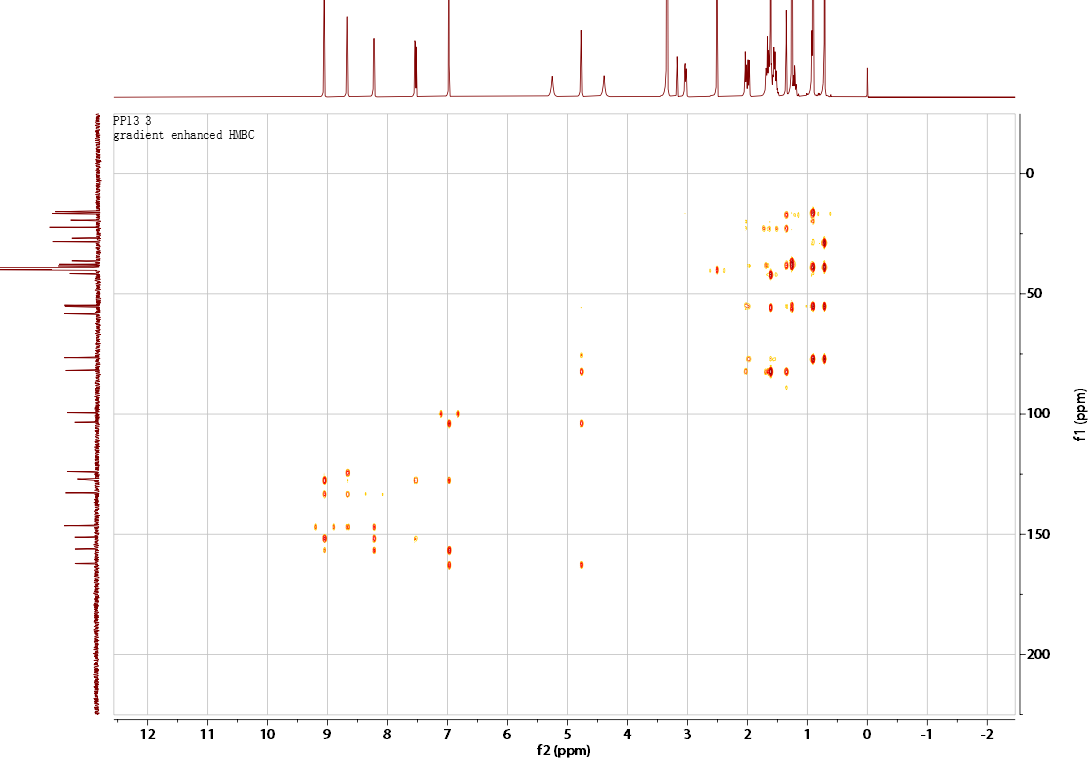


The HMBC spectrum of compound **3** in DMSO-*d*_6_


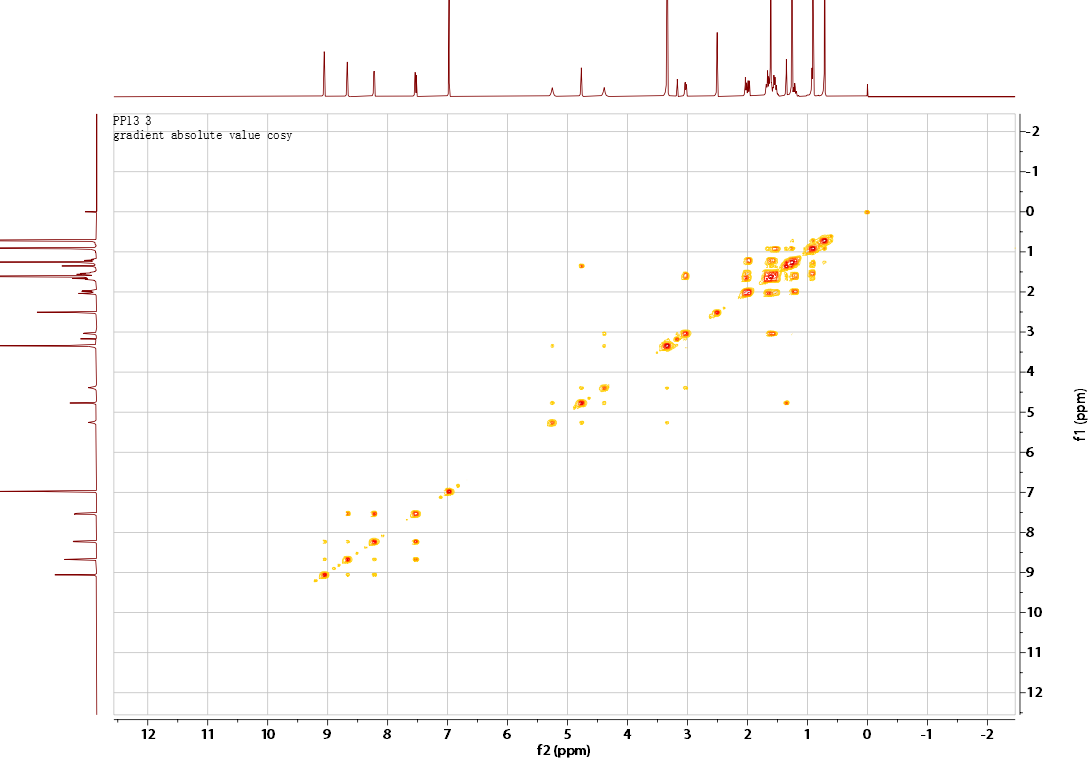


The ^1^H-^1^H COSY spectrum of compound **3** in DMSO-*d*_6_


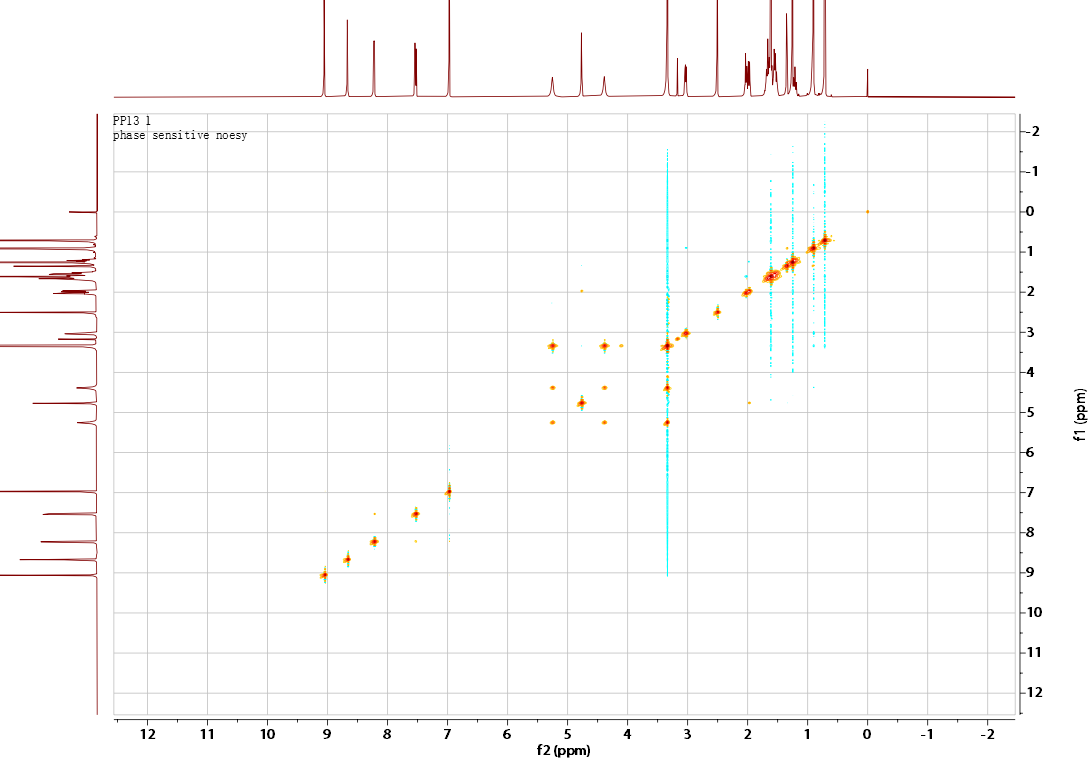


The NOESY spectrum of compound **3** in DMSO-*d*_6_


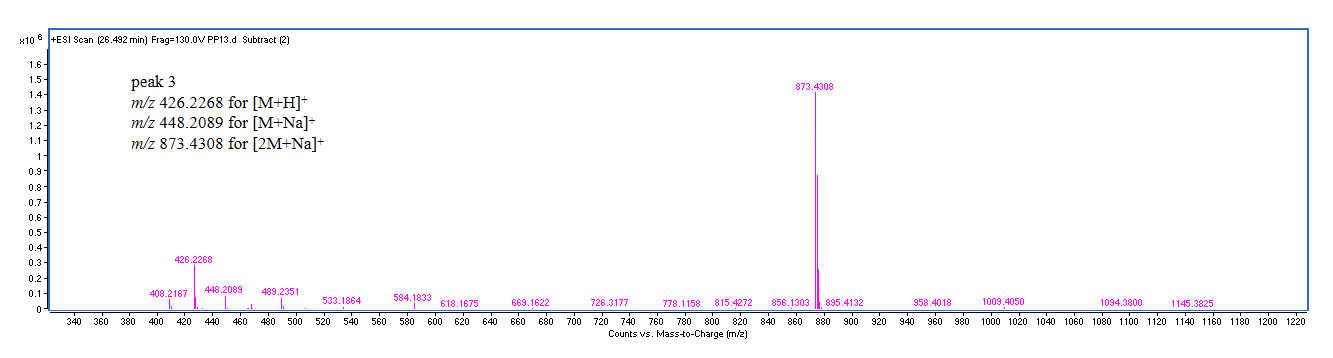


The TOF-ESI-MS spectrum of compound **3**

# Figure S4. The 1D-NMR, 2D-NMR, and TOF-ESI-MS spectra of compound 3.


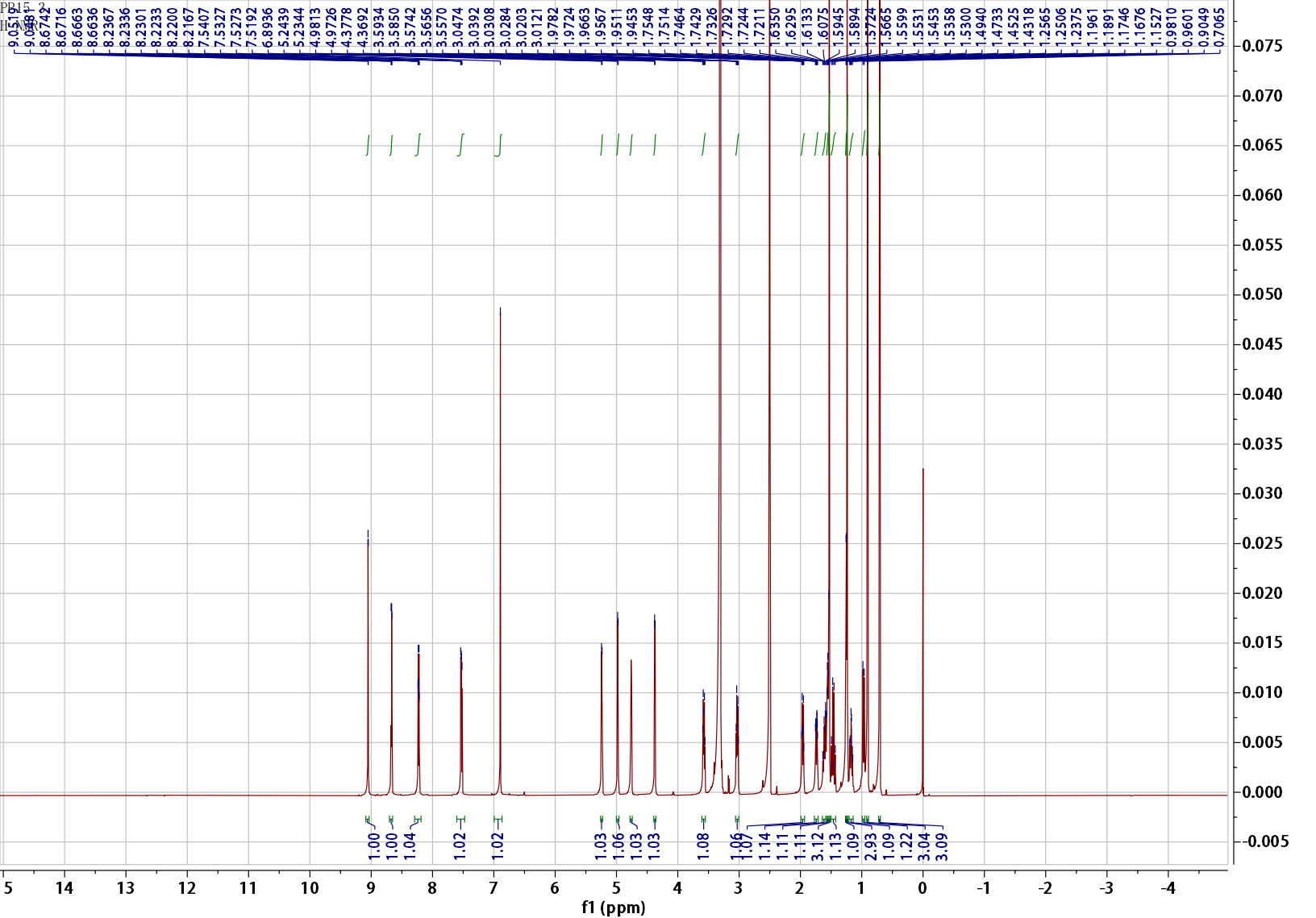


The ^1^H NMR spectrum of compound **4** in DMSO-*d*_6_


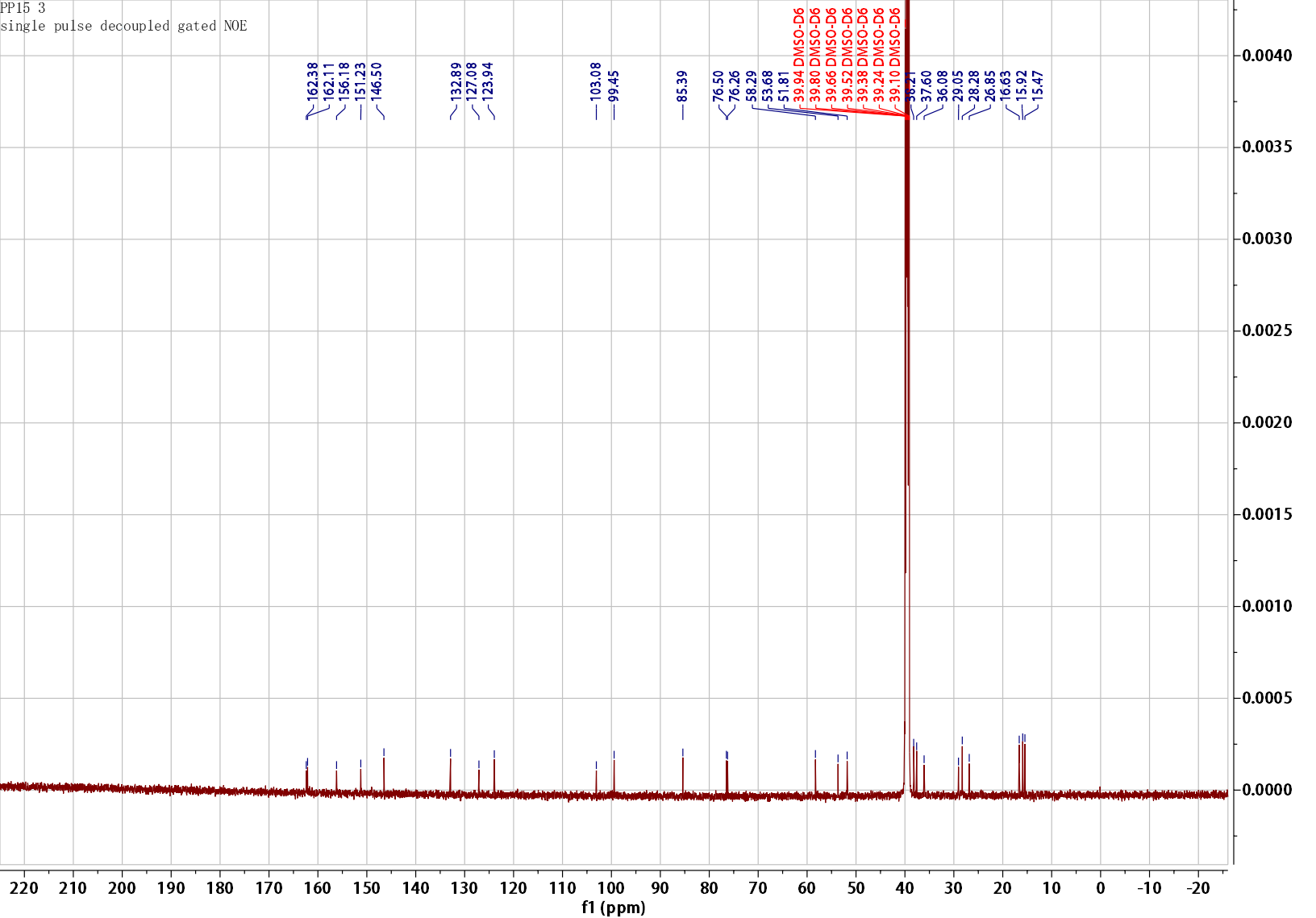


The ^13^C NMR spectrum of compound **4** in DMSO-*d*6


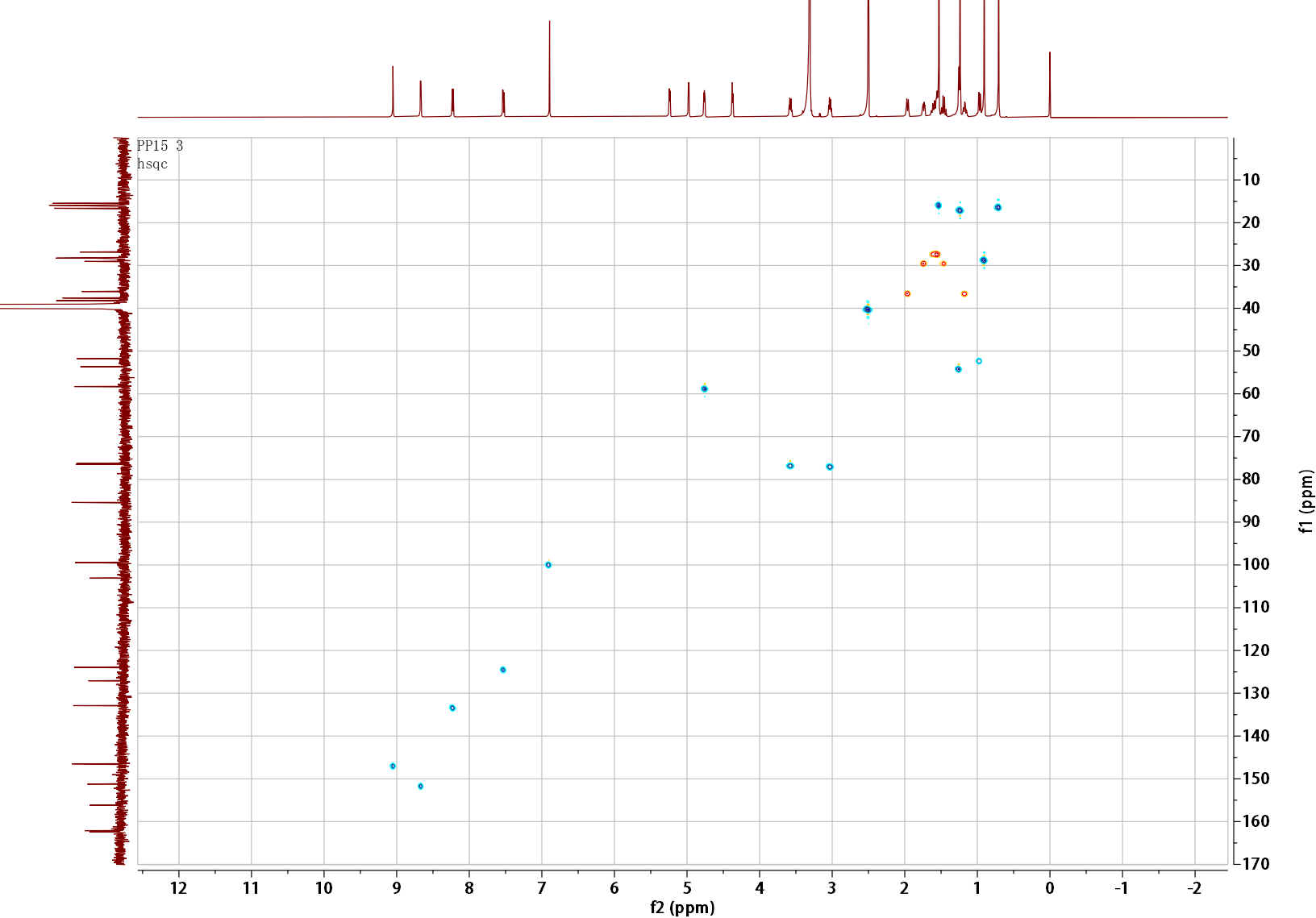


The HSQC spectrum of compound **4** in DMSO-*d*_6_


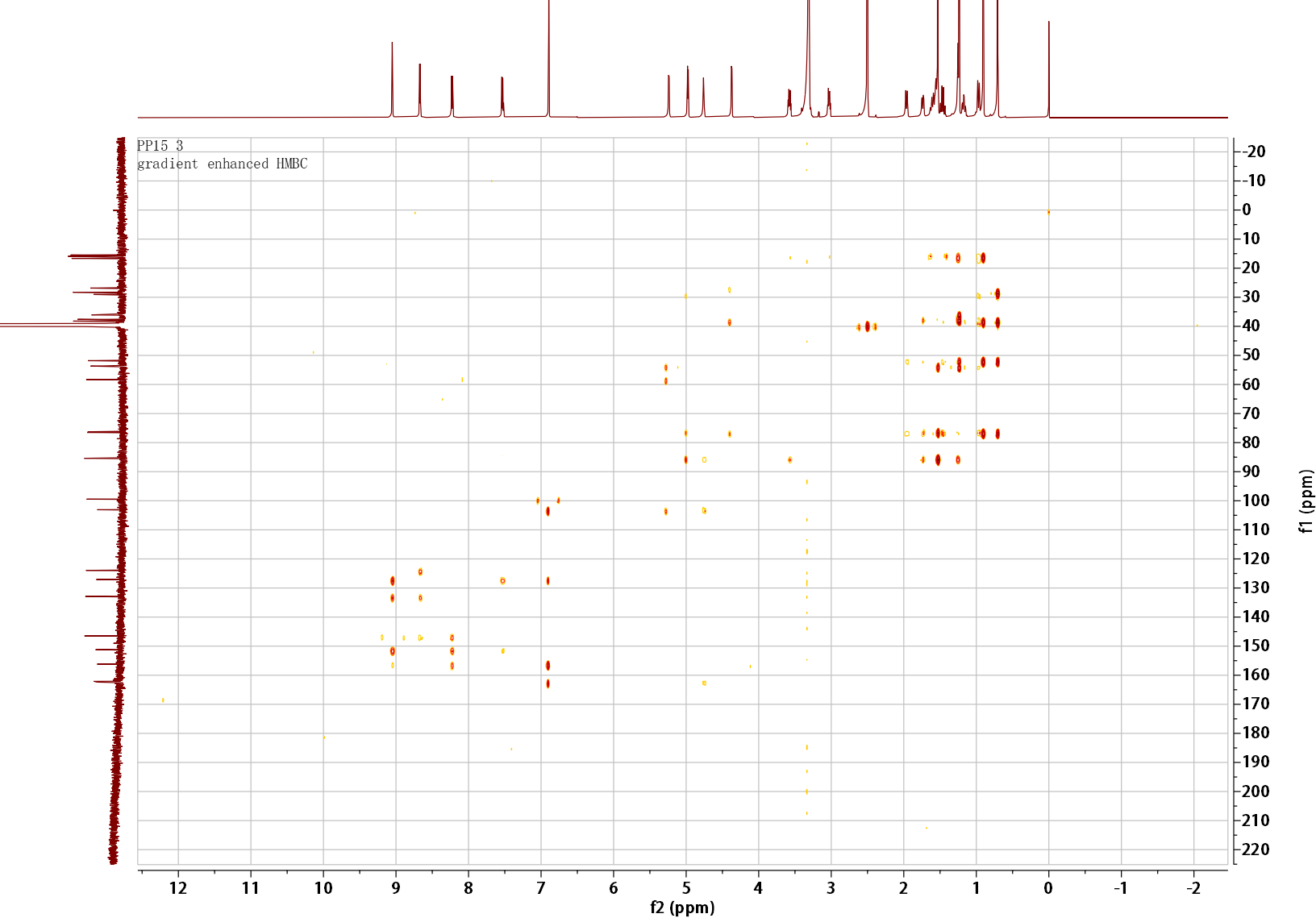


The HMBC spectrum of compound **4** in DMSO-*d*_6_


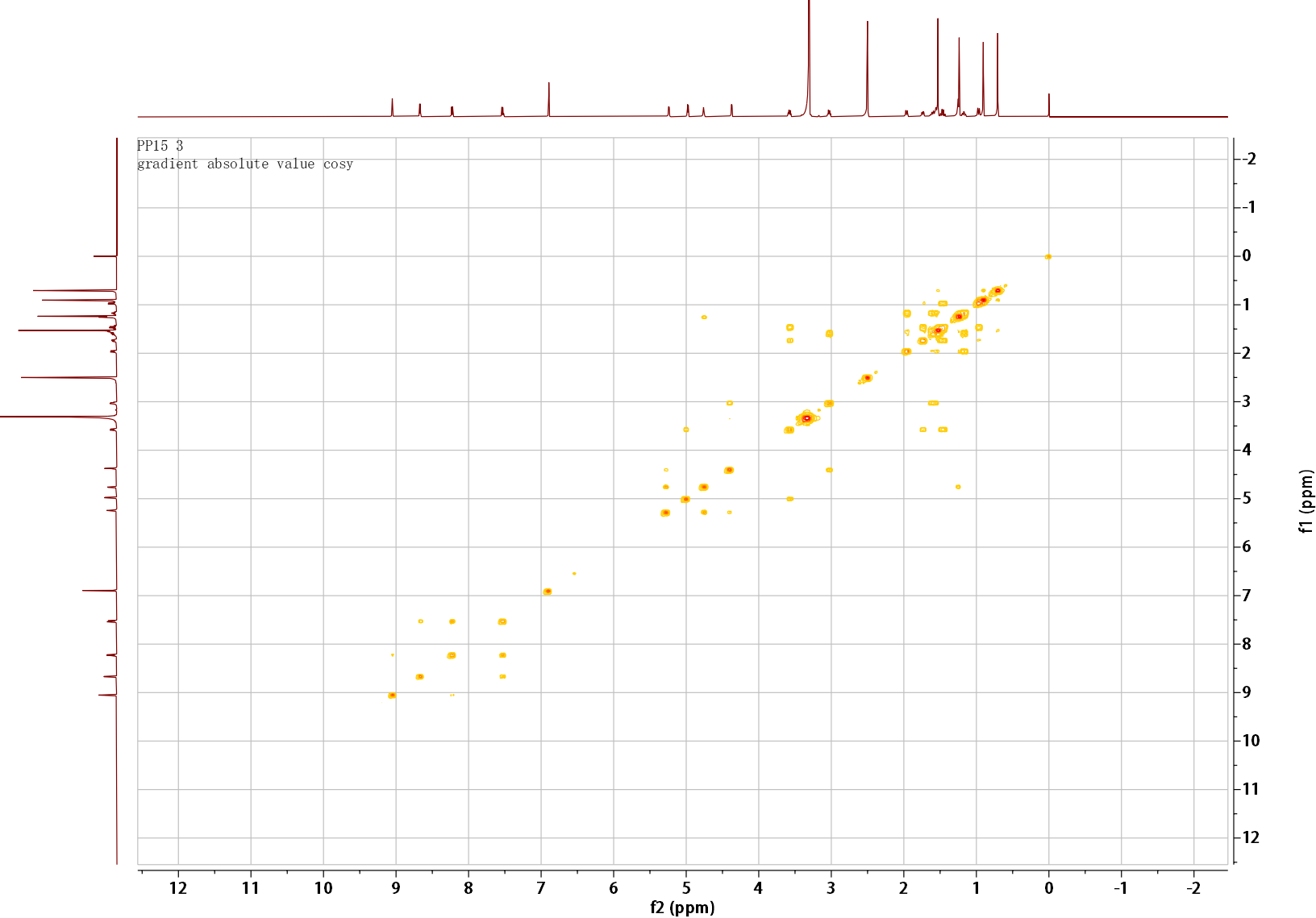


The ^1^H-^1^H COSY spectrum of compound **4** in DMSO-*d*_6_


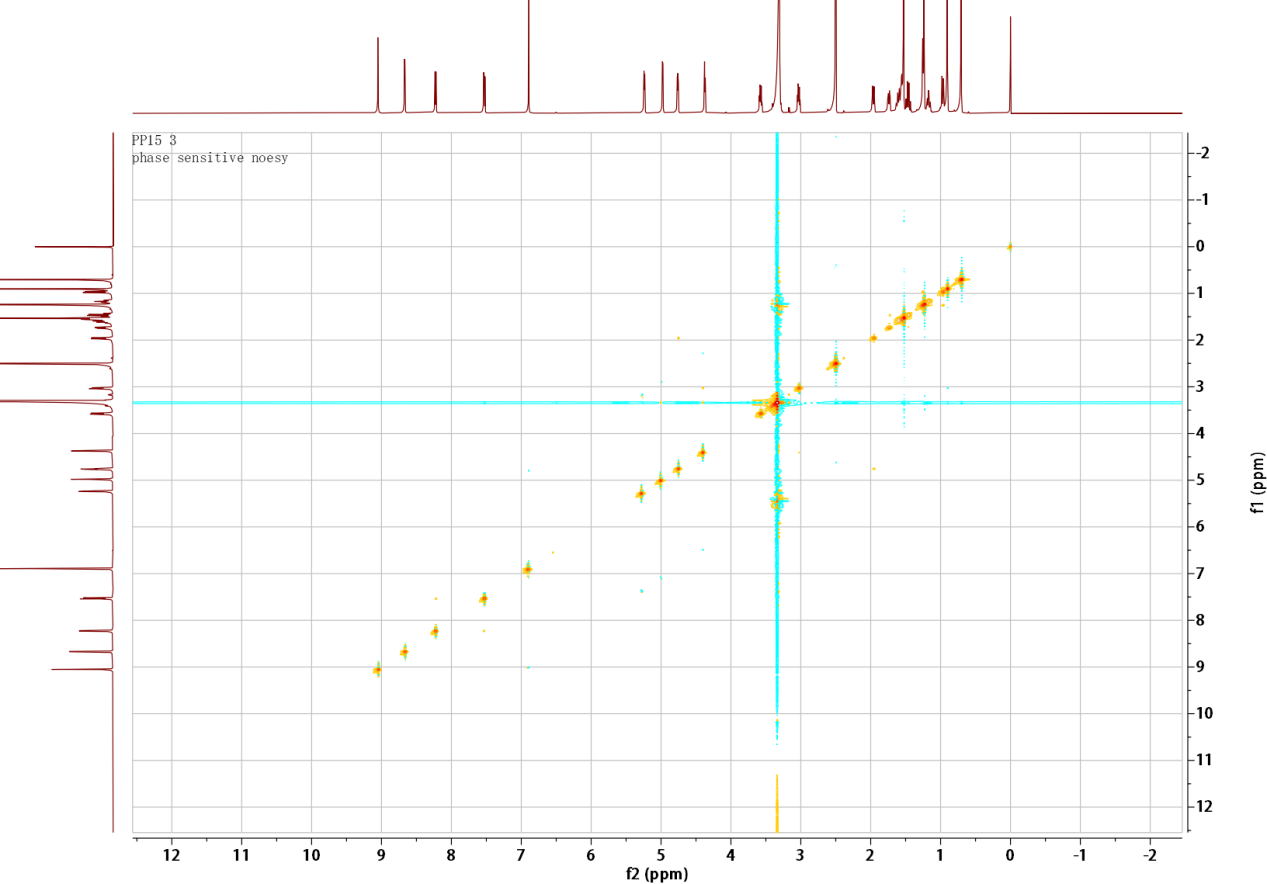


The NOESY spectrum of compound **4** in DMSO-*d*_6_


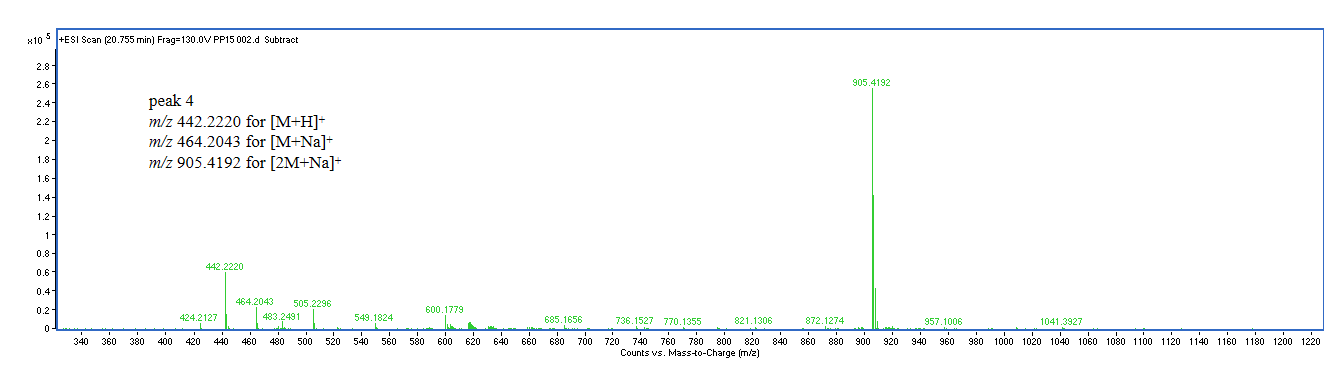


The TOF-ESI-MS spectrum of compound **4**

# Figure S5. The 1D-NMR, 2D-NMR, and TOF-ESI-MS spectra of compound 4.


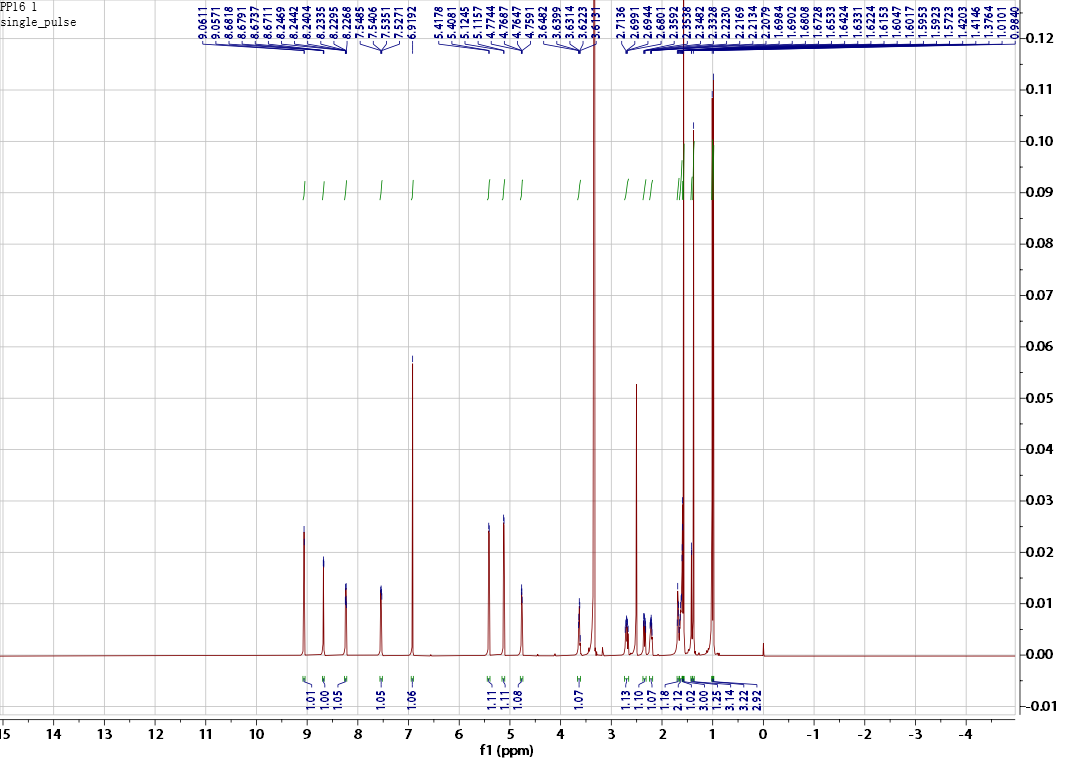


The ^1^H NMR spectrum of compound **5** in DMSO-*d*_6_


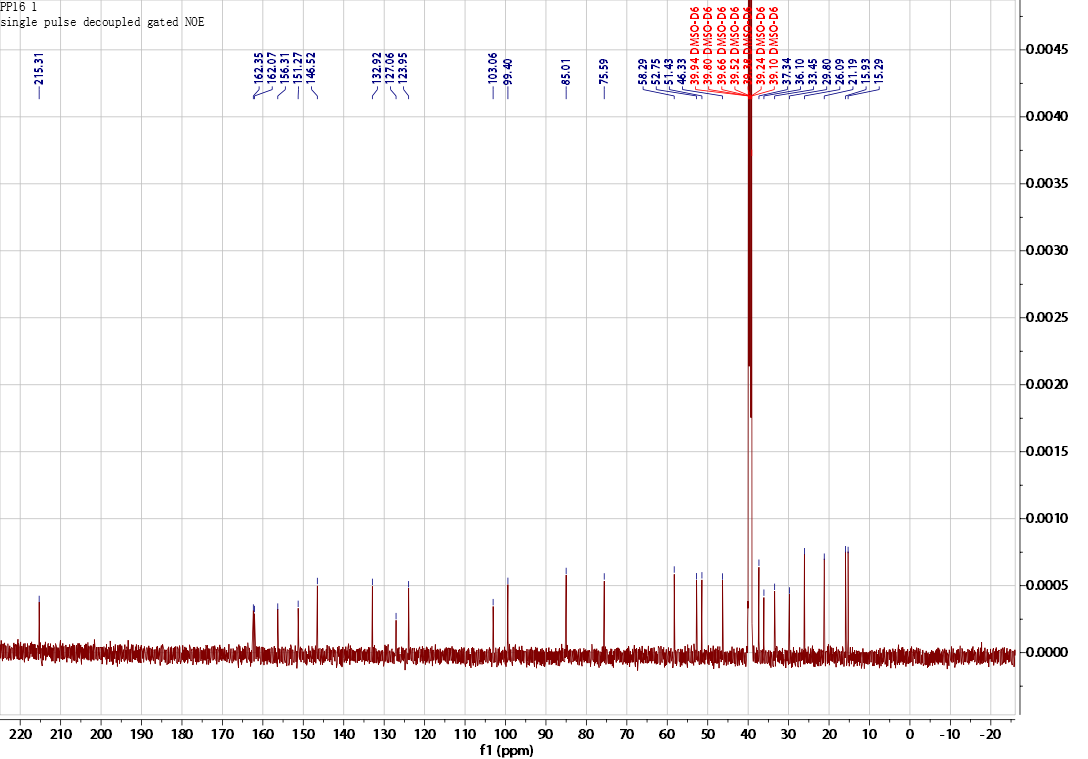


The ^13^C NMR spectrum of compound **5** in DMSO-*d*6


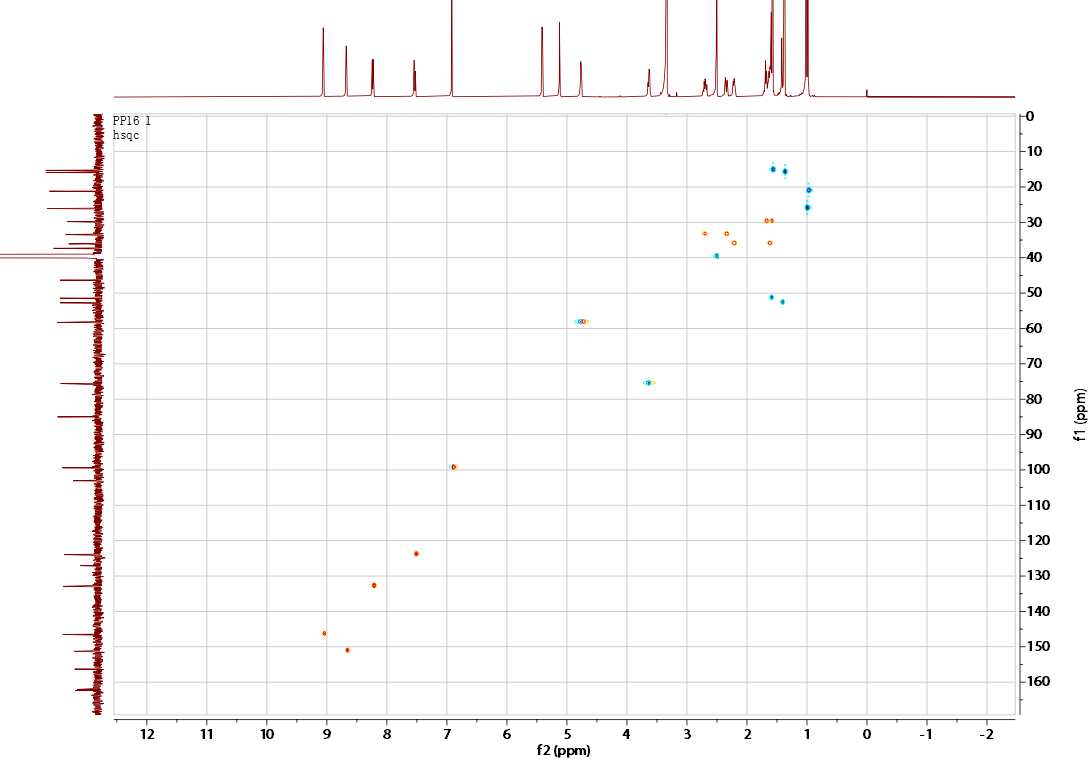


The HSQC spectrum of compound **5** in DMSO-*d*_6_


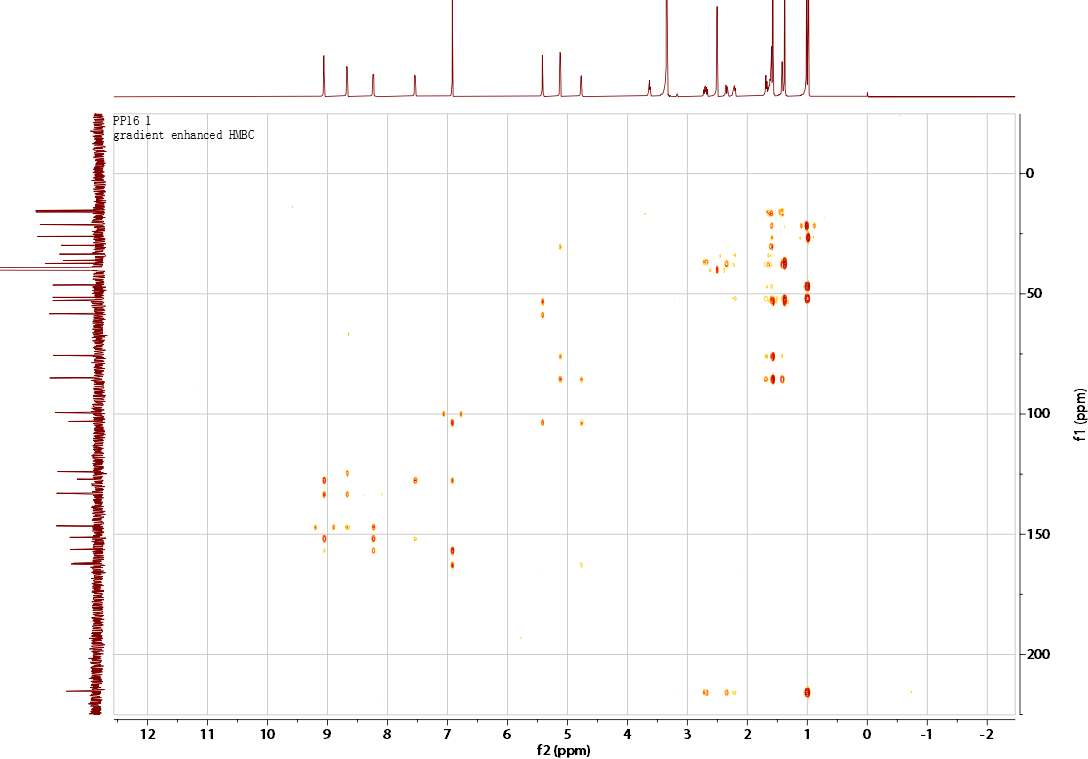


The HMBC spectrum of compound **5** in DMSO-*d*_6_


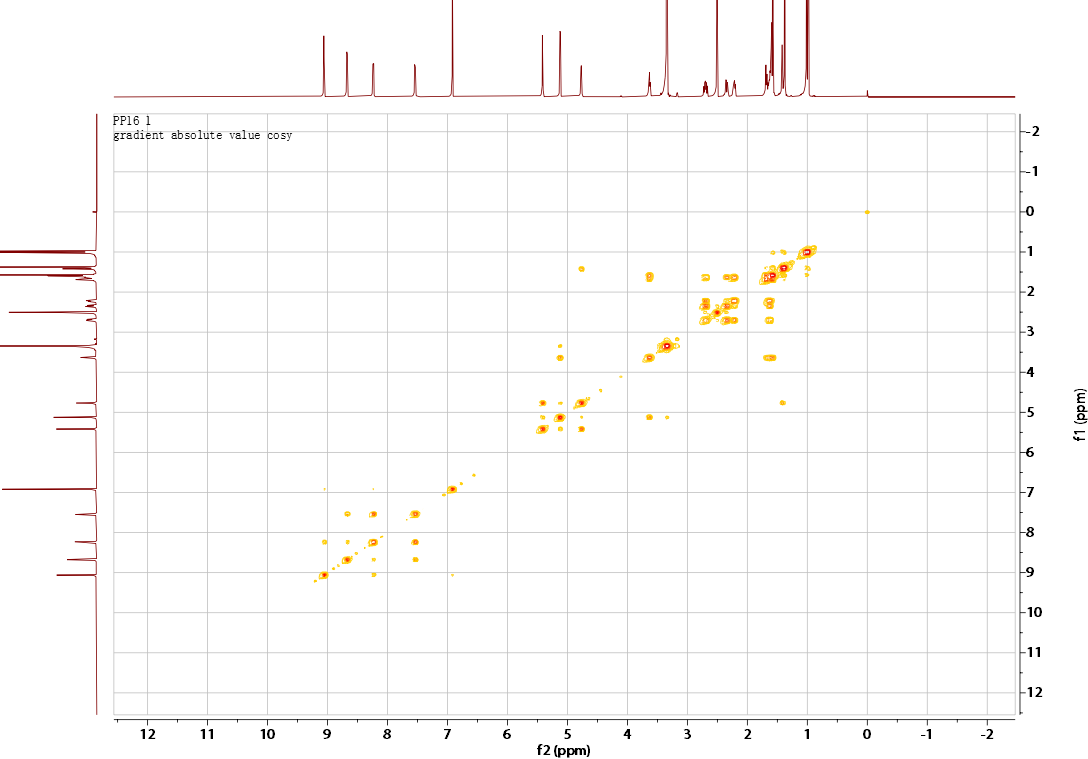


The ^1^H-^1^H COSY spectrum of compound **5** in DMSO-*d*_6_


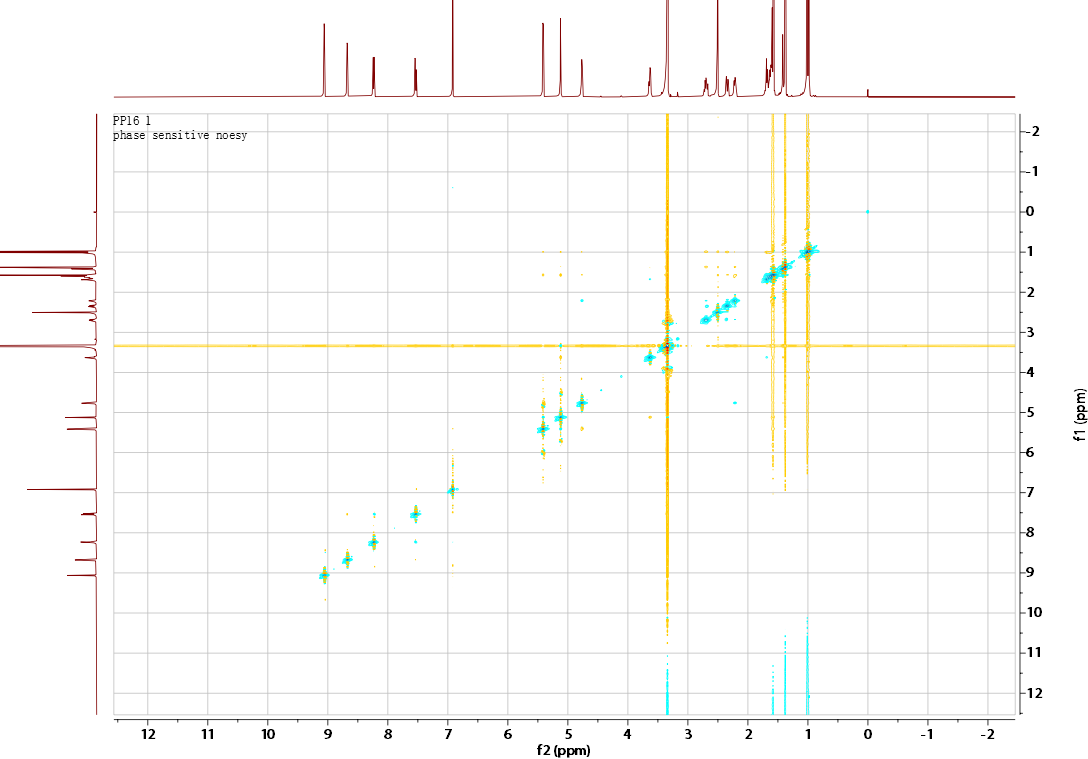


The NOESY spectrum of compound **5** in DMSO-*d*_6_


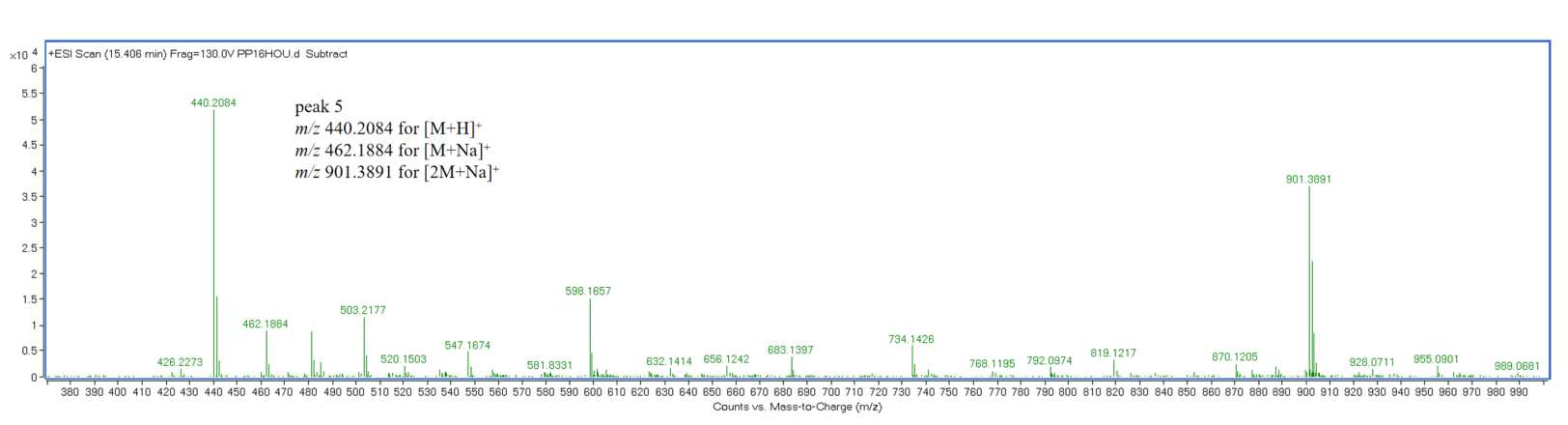


The TOF-ESI-MS spectrum of compound **5**

# Figure S6. The 1D-NMR, 2D-NMR, and TOF-ESI-MS spectra of compound 5.

# Supplementary References

Chiang YM, Ahuja M, Oakley CE, Entwistle R, Asokan A, Zutz C, Wang CCC, Oakley BR. 2016. Development of genetic dereplication strains in *Aspergillus nidulans* results in the discovery of aspercryptin. Angew Chem Int Ed Engl. 55(5):1662-5. doi: 10.1002/anie.201507097.

Osherov N, Kontoyiannis DP, Romans A, May GS. 2001. Resistance to itraconazole in *Aspergillus nidulans* and *Aspergillus fumigatus* is conferred by extra copies of the *A. nidulans* P-450 1α-demethylase gene, *pdmA*. J Antimicrob Chemother. 48(1):75–81. doi: 10.1093/jac/48.1.75.

Yin WB, Chooi YH, Smith AR, Cacho RA, Hu Y, White TC, Tang Y. 2013. Discovery of cryptic polyketide metabolites from dermatophytes using heterologous expression in *Aspergillus nidulans*. ACS Synth Biol. 2(11):629-34. doi: 10.1021/sb400048b.
